# Supplementary material for: High‐throughput screening identifies suppressors of mitochondrial fragmentation in OPA1 fibroblasts
Source: EMBO Mol Med. 2021 May 20;13(6):e13579. doi: 10.15252/emmm.202013579 (PMC8185549; doi:10.15252/emmm.202013579)
Supplement: Supplementary file 1 — Appendix [file EMMM-13-e13579-s007.pdf]

High-throughput screening identifies suppressors of mitochondrial fragmentation in *OPA1* fibroblasts

Emma CRETIN[1,2] Priscilla LOPES[1], Elodie VIMONT[1], Takashi TATSUTA[3], Thomas LANGER[3,4], Anastasia GAZI[5], Martin SACHSE[5], Patrick YU-WAI-MAN[6-9], Pascal REYNIER[10,11], Timothy WAI[1,2]*

APPENDIX

Contents: Appendix Figures S1-S8

Appendix Figure S1: Mitochondrial morphology quantification in fibroblasts and Western blot analysis of human fibroblasts

Appendix Figure S2: STRING analysis of Mitome screen hits identified in control human fibroblasts.

Appendix Figure S3: STRING analysis of Mitome screen hits discovered in DOA+ fibroblasts and and validation of PGS1 downregulation in OPA1-deficient HeLa cells.

Appendix Figure S4:Mitochondrial morphology and stress-induced hyperfusion analysis in Opa1-deficient MEFs.

Appendix Figure S5: DRP1 recruitment assays in MEFs

Appendix Figure S6: Membrane potential and kinetic imaging of fission and hyperfusion in MEFs

Appendix Figure S7: Determination of cardiolipin profiling and phosphatidic acid accumulation in MEFs.

Appendix Figure S8: Kinetic cell death imaging in MEFs triggered by etoposide

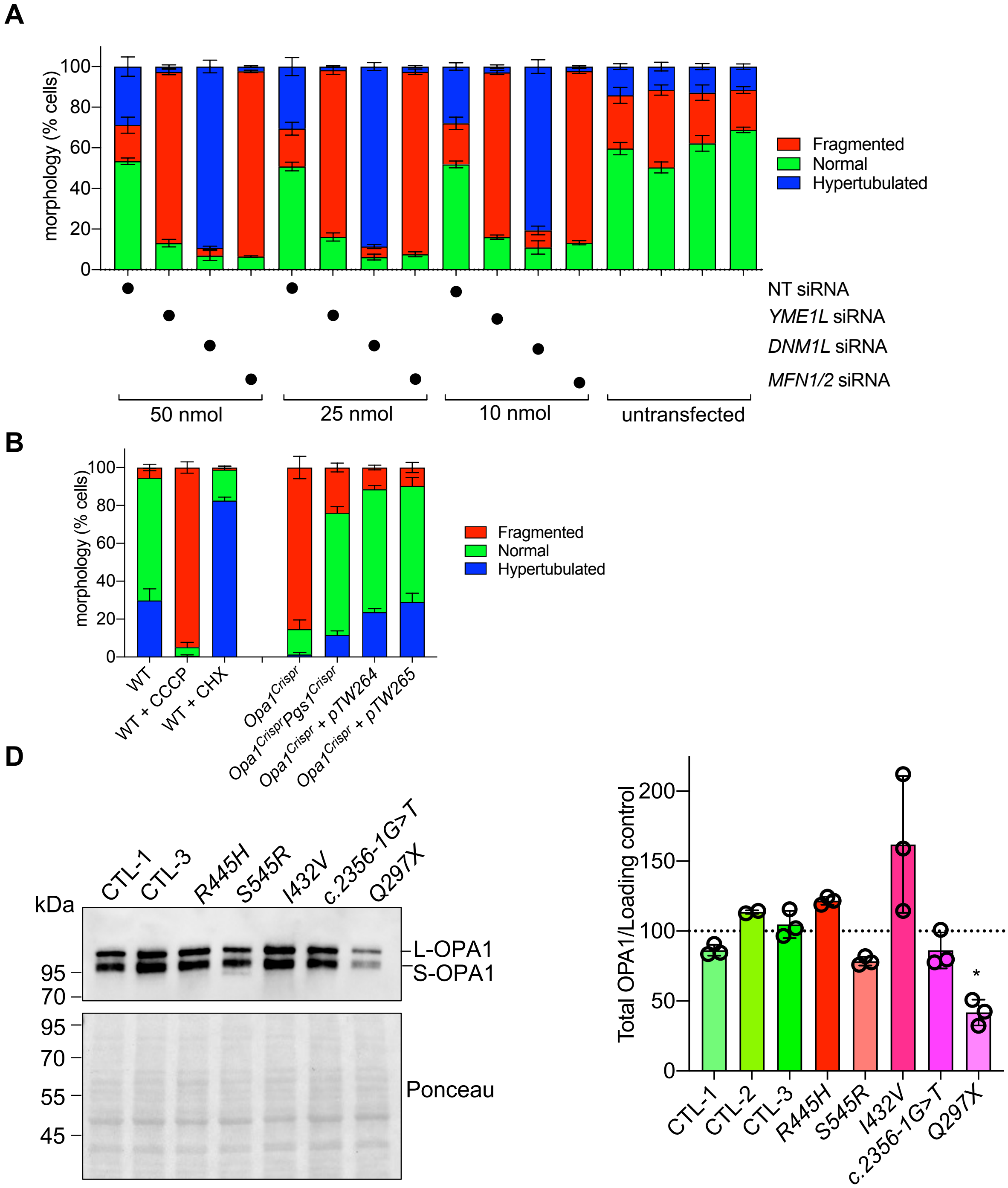

Appendix Figure S1: Mitochondrial morphology quantification in fibroblasts and Western blot analysis of human fibroblasts

(A) Mitochondrial morphology quantification of control (CTL-1) fibroblasts treated with indicated siRNAs at the indicated doses for 72 hours. Supervised ML training performed on cells with fragmented (*OPA1* siRNA), normal (non-targeting NT siRNA), and hypertubulated (*DNM1L* siRNA) mitochondria. Data represent mean ± SD of 3 independent experiments, (1005 to 2803 cells per cell line per replicate), One-way ANOVA. **(B)** Supervised ML performed on mitochondrial morphologies generated by chemical manipulation (fragmented, CCCP, hypertubulated, cycloheximide (CHX)) classify Opa1-deficient MEFs (*Opa1*^{Crispr}) from Figure 3E before and after stable functional complementation with of Opa1s1-Myc (plasmid pTW264, Dataset EV5) or Opa1s7-Myc (plasmid pTW265, Dataset EV5) and *Opa1*^{Crispr} MEFs additional deleted of *Pgs1* from Figure 3E (*Opa1*^{Crispr}*Pgs1*^{Crispr}). Data represent mean ± SD of 3-6 independent wells (1693 to 2554 cells per cell line), One-way ANOVA (% normal).

(C) Equal amounts of protein extracted from control and (CTL1, 2, 3) and DOA+ patient fibroblasts were separated by SDS-PAGE, immunoblotted with anti-OPA1 antibody, and quantified by densitometry relative to Ponceau stain. Data represent mean ± SD of three independent experiments with the exception of CTL-2, which was measured twice, One-way non-parametric ANOVA (Kruskal-Wallis) (relative to mean of CTL1, 2 and 3 combined); *p>0.05.

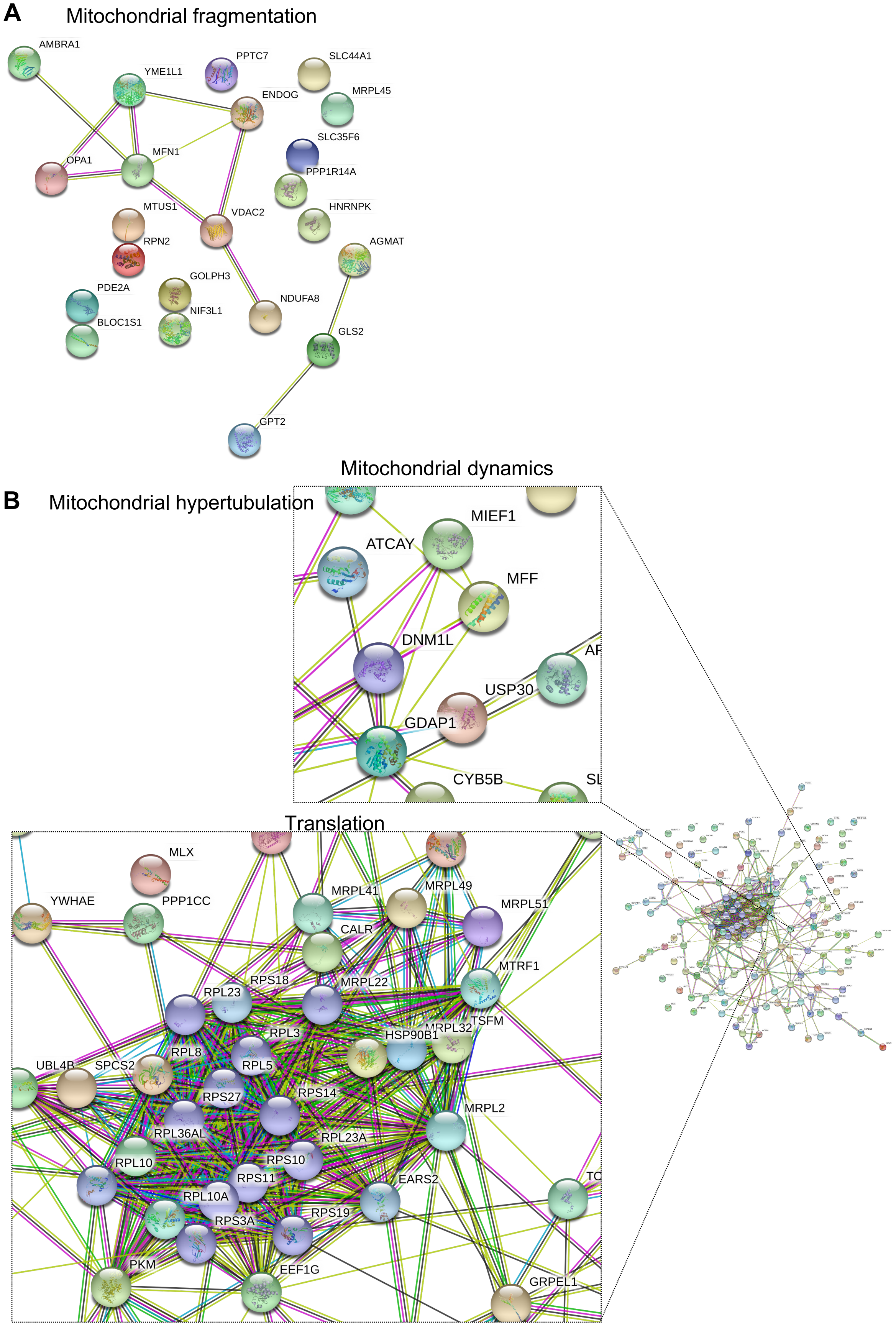

Appendix Figure S2: STRING analysis of Mitome screen hits identified in control human fibroblasts.

(A) String analysis clustering of 22 candidate genes that stimulate mitochondrial fragmentation upon depletion (Dataset EV2)

(B) String analysis clustering of 145 candidate genes that stimulate mitochondrial hypertubulation upon depletion (Dataset EV2), including clusters related to mitochondrial dynamics (top inset) and protein translation (bottom inset), (Dataset EV2).

Appendix Figure 3

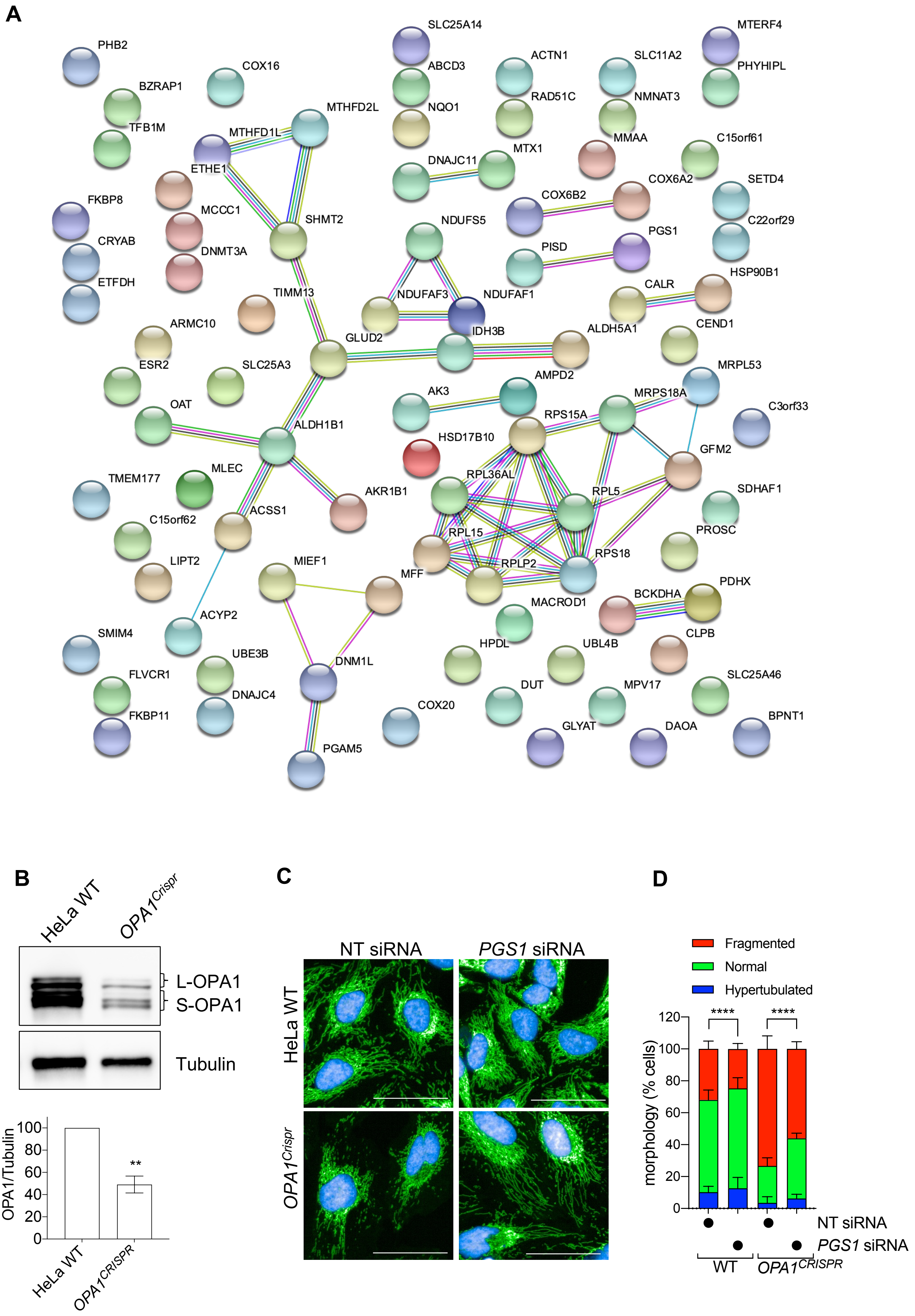

Appendix Figure S3: STRING analysis of Mitome screen hits and validation of PGS1 downregulation in OPA1-deficient HeLa cells.

(A) String analysis clustering of 91 candidate genes that rescued mitochondrial fragmentation in *OPA1*^{S545R} fibroblasts upon depletion (Dataset EV3).

(B) Equal amounts of protein extracted from WT and *OPA1*^{CRISPR} HeLa were separated by SDS-PAGE, immunoblotted with OPA1 antibody and quantified by densitometry relative to tubulin. Data represent mean ± SEM of three independent experiments. Unpaired t-test; **p<0.01.

(C) Representative confocal images of wild type (WT) and *OPA1*^{CRISPR} HeLa cells treated with indicated siRNAs for 72 hours. Live imaging of mitochondria labelled with TMRE (Mitochondria, green), and NucBlue (Nuclei, blue). Scale bar=50μm.

(D) Supervised ML mitochondrial morphology quantification of (m) using WT HeLa cells treated with *OPA1* siRNA (fragmented), NT siRNA (normal), or *DNM1L* siRNA (hypertubulated) training sets. Data represent mean ± SD of 8 replicates, One-way ANOVA; ****p <0.0001.

Appendix Figure 4

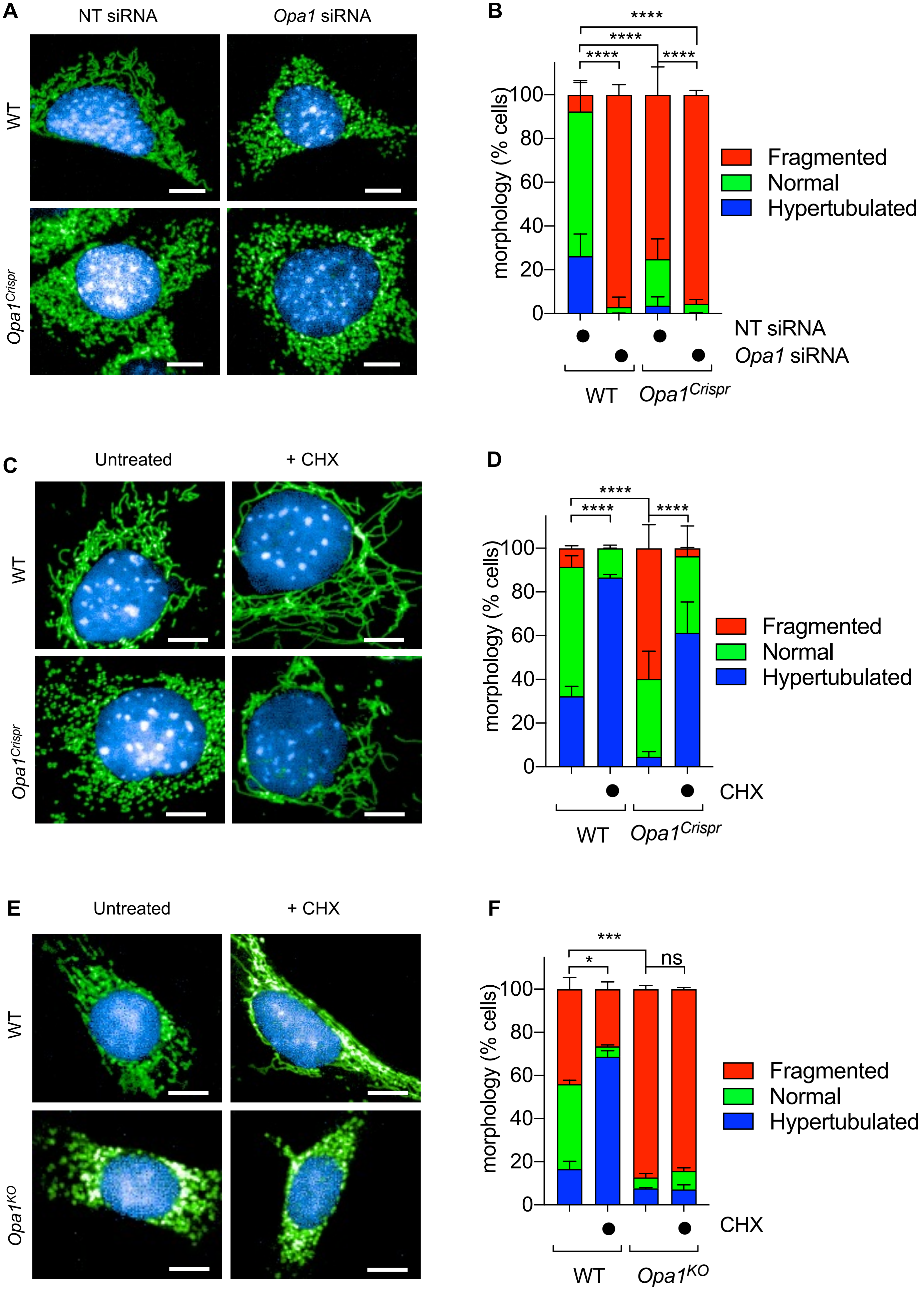

Appendix Figure S4: Mitochondrial morphology and stress-induced hyperfusion analysis in Opa1-deficient MEFs.

(A) Representative confocal images of wild type (WT) and *Opa1*^{Crispr} MEFs treated with NT or *Opa1* siRNA for 72 hours. Live imaging of mitochondria (mitoYFP, green) and nuclei (NucBlue, blue). Scale bar=10µm.
(B) Mitochondrial morphology quantification of (i) using WT MEFs treated with *Opa1* siRNA (fragmented), NT siRNA (normal), or *Dnm1l* siRNA (hypertubulated) training sets. Data represent mean ± SD of three independent experiments, (550-822 cells per cell line), One-way ANOVA (% fragmented) ; ****$p < 0.0001$.
(C) Representative confocal images of wild type (WT) and *Opa1*^{Crispr} MEFs treated with 50 µM cycloheximide (CHX) for 3 hours. Live imaging of mitochondria (mitoYFP, green) and nuclei (NucBlue, blue). Scale bar=10µm.
(D) Mitochondrial morphology quantification of (k) using WT MEFs treated with *Opa1* siRNA (fragmented), NT siRNA (normal), or *Dnm1l* siRNA (hypertubulated) training sets. Data represent mean ± SD of three independent experiments, (547-822 cells per cell line), One-way ANOVA (% fragmented) ; ****$p < 0.0001$.
(E) Representative confocal images of wild type (WT) and *Opa1*^{KO} MEFs treated with 50 µM cycloheximide (CHX) for 3 hours. Live imaging of mitochondria (mitoYFP, green) and nuclei (NucBlue, blue). Scale bar=10µm.
(F) Mitochondrial morphology quantification of (m) using WT MEFs treated with *Opa1* siRNA (fragmented), NT siRNA (normal), or *Dnm1l* siRNA (hypertubulated) training sets. Data represent mean ± SD of two independent experiments, (638-1063 cells per cell line) One-way ANOVA (% hypertubulated) ; *$p < 0.05$, ***$p < 0.001$, ns; not significant.

Appendix Figure 5

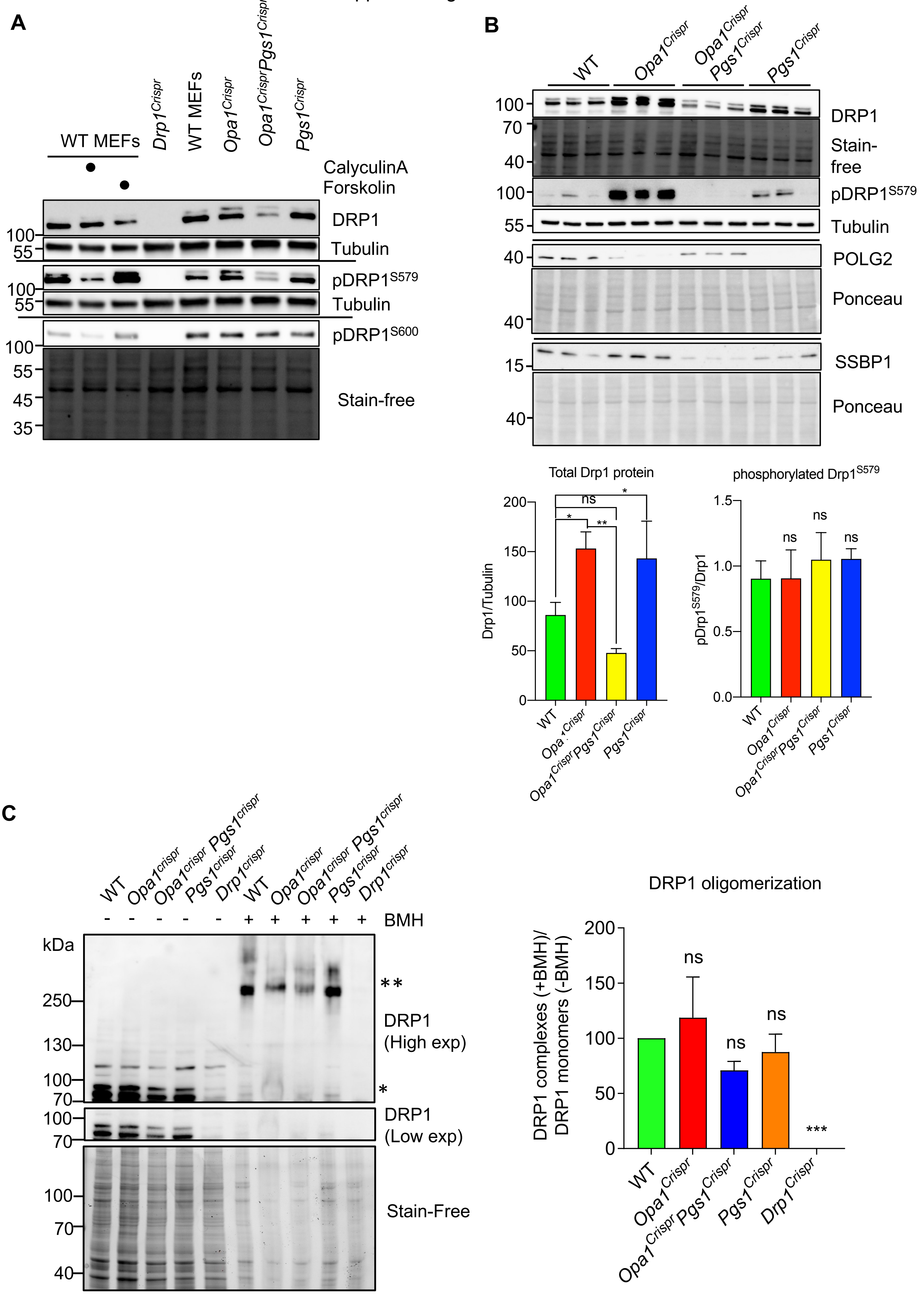

Appendix Figure S5: Drp1 recruitment assays in MEFs

(A) Equal amounts of protein extracted from MEFs treated with CalyculinA or Forskolin, where indicated, were separated by SDS-PAGE, immunoblotted with the indicated antibody, Stain-Free or Ponceau.
(B) Equal amounts of protein extracted from MEFs were separated by SDS-PAGE, immunoblotted with the indicated antibody and quantified by densitometry relative to Stain-Free or Ponceau. Data represent mean ± SD of three independent experiments, One-way ANOVA; *p < 0.05, **p < 0.01, ns; not significant.
(C) Equal amount of post-nuclear supernatant +/- cross-linked with 10mM BMH from MEFs of the indicated genotypes were separated by SDS-PAGE and immunoblotted with DRP1 antibody. * and ** indicate DRP1 monomers and DRP1 complexes, respectively. The ration of DRP1 complexes over DRP1 monomers was quantified by densitometry relative to Stain-Free. Data represent mean ± SD of 3 independent experiments, One-way ANOVA; ***p < 0.001, ns; not significant

Appendix Figure 6

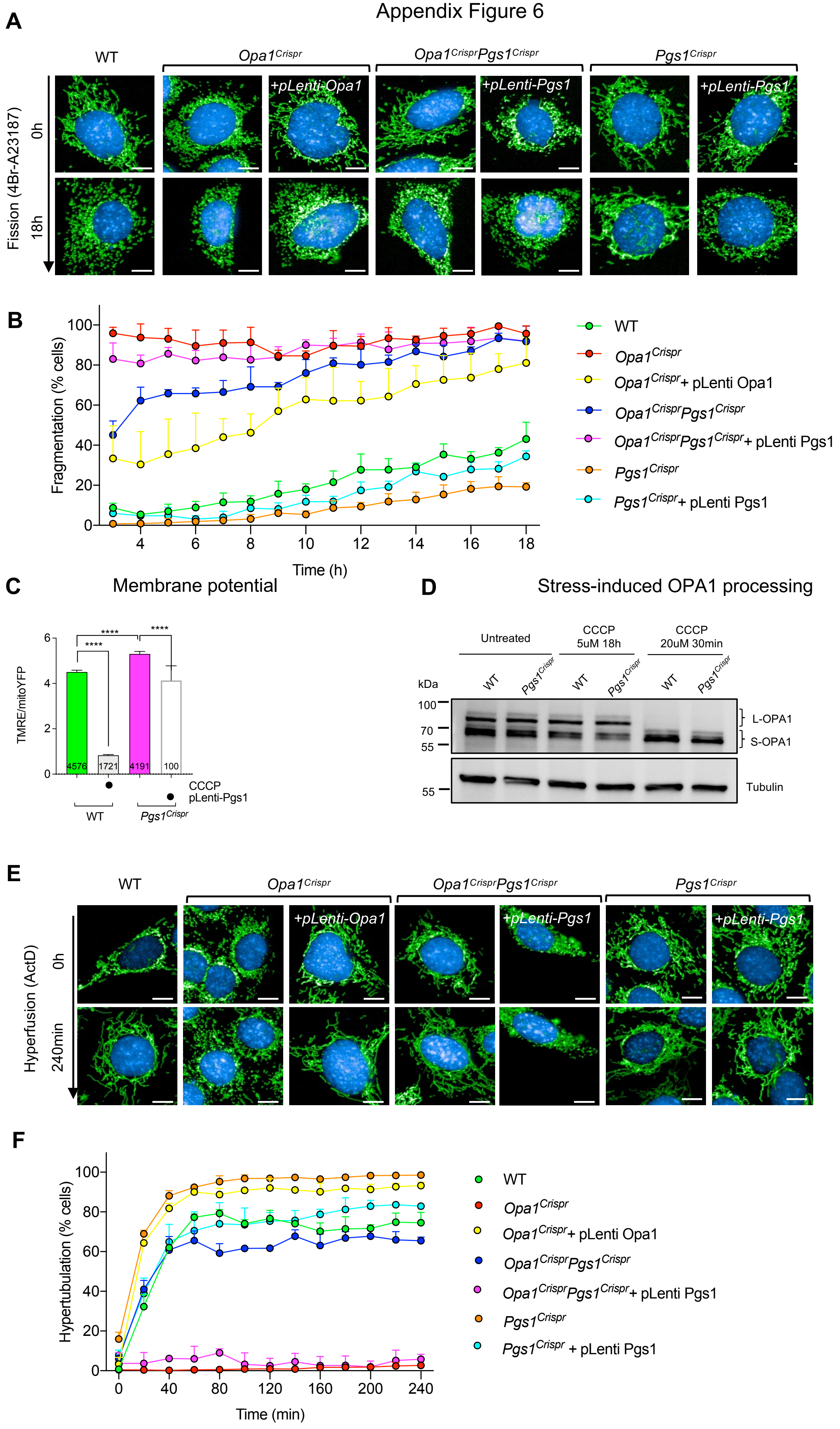

Appendix Figure S6: Membrane potential and kinetic imaging of fission and hyperfusion in MEFs

(A) Representative confocal images of live cell imaging of MEFs of the indicated genotypes subjected to fission with 4Br-A23187 for the indicated time points. Images were captured every hour for 18 hours. Scale bar=10μm.
(B) Mitochondrial morphology quantification of (A) using WT MEFs treated with 5 μM CCCP for 18h (fragmented), untreated (normal), or treated with 10 μM CHX for 9h (hypertubulated) training sets. Data represent mean ± SD of 3 independent experiments, (355-1196 cells per cell line) One-way ANOVA.
(C) Quantification of mitochondrial membrane potential (TMRE/mitoYFP) by confocal microscopy in WT and *Pgs1^{Crispr}* MEFs treated as indicated. Number of analyzed cells indicated within bar. Data represent mean of all the cells ± SEM of four independent experiments, One-way ANOVA; ****$p < 0.0001$.
(D) Equal amounts of protein extracted from WT and mutant MEFs treated with CCCP at the indicated concentrations and durations where indicated were separated by SDS-PAGE, immunoblotted with indicated antibodies.
(E) Representative confocal images of live cell imaging of MEFs of the indicated genotypes subjected to stress-induced mitochondrial hyperfusion (SiMH) with 0.5 μM ActD for the indicated time points. Images were captured every 20 minutes for 240 minutes. Scale bar=10μm.
(F) Mitochondrial morphology quantification of using WT MEFs treated with 5 μM CCCP for 18h (fragmented), untreated (normal), or treated with 10 μM CHX for 9h (hypertubulated) training sets. Data represent mean ± SD of 3 independent experiments, (262-1123 cells per cell line) One-way ANOVA.

Appendix Figure 7

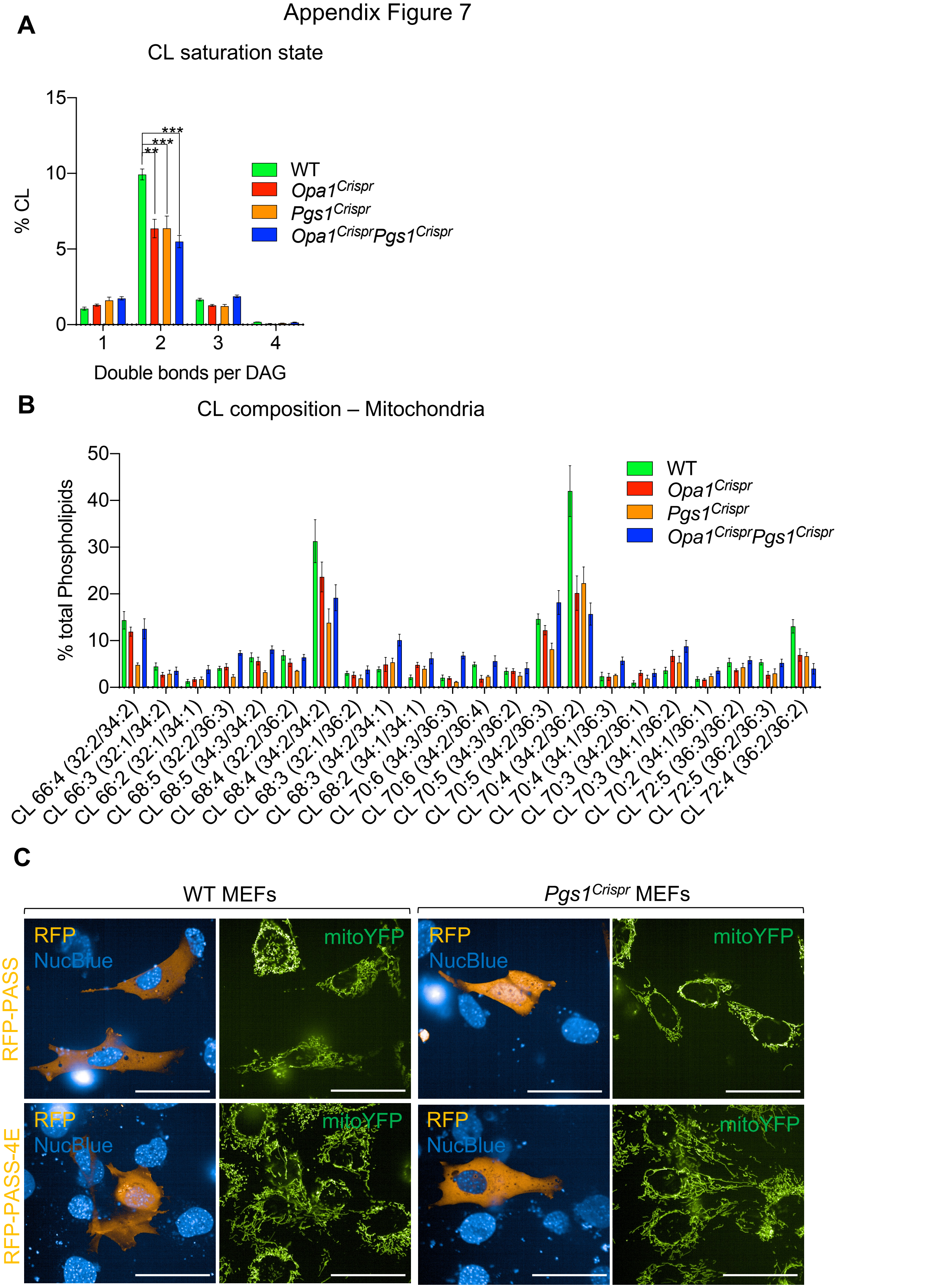

Appendix Figure S7: Determination of cardiolipin profiling and phosphatidic acid accumulation in MEFs.

(A) Cardiolipin (CL) saturation state (carbon double bonds per DAG molecule) determined from purified mitochondria isolated from MEFs of the indicated genotypes. Data represent mean ± SD of four independent experiments, One-way ANOVA;$^{**}p < 0.01$, $^{***}p < 0.001$.

(B) CL acyl chain composition determined from purified mitochondria isolated from MEFs of the indicated genotypes. Data represent mean ± SD of four independent experiments.

(C) Representative confocal images of wild type (WT) and *Pgs1*^{Crispr} MEFs transfected with wild type (RFP-PASS) or mutant (RFP-PASS-4E) PA sensors 48 hours. Live imaging of mitochondria labelled with mitoYFP (green), RFP (orange) and NucBlue (Nuclei, blue) revealed no recruitment of PA sensors to mitochondria. Scale bar=50µm.

Appendix Figure 8

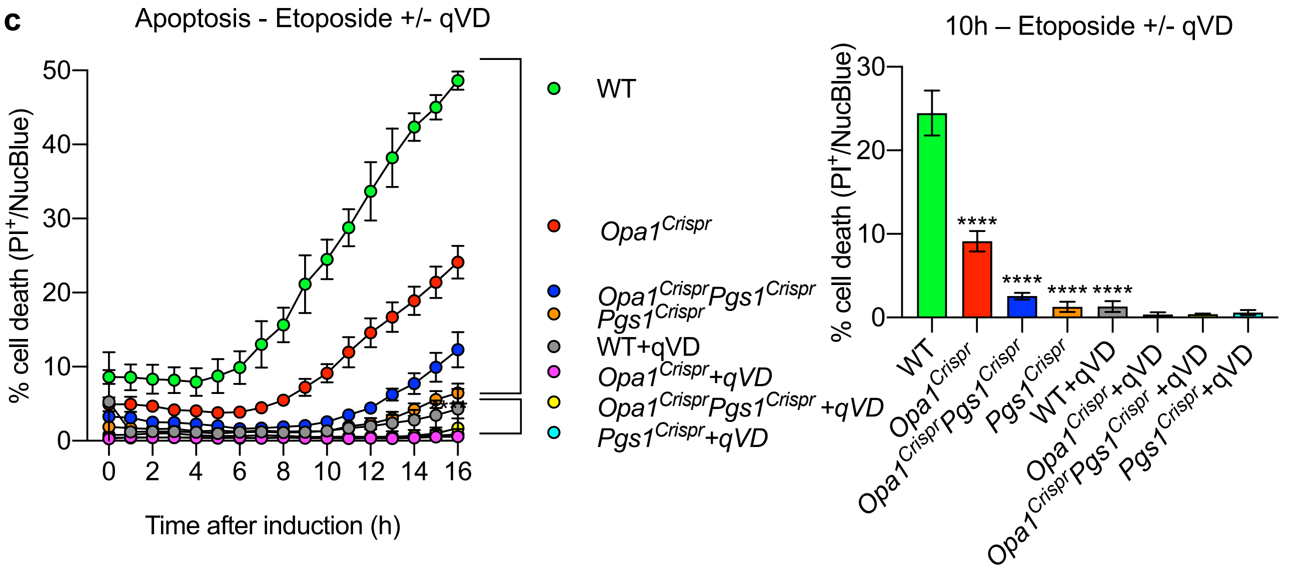

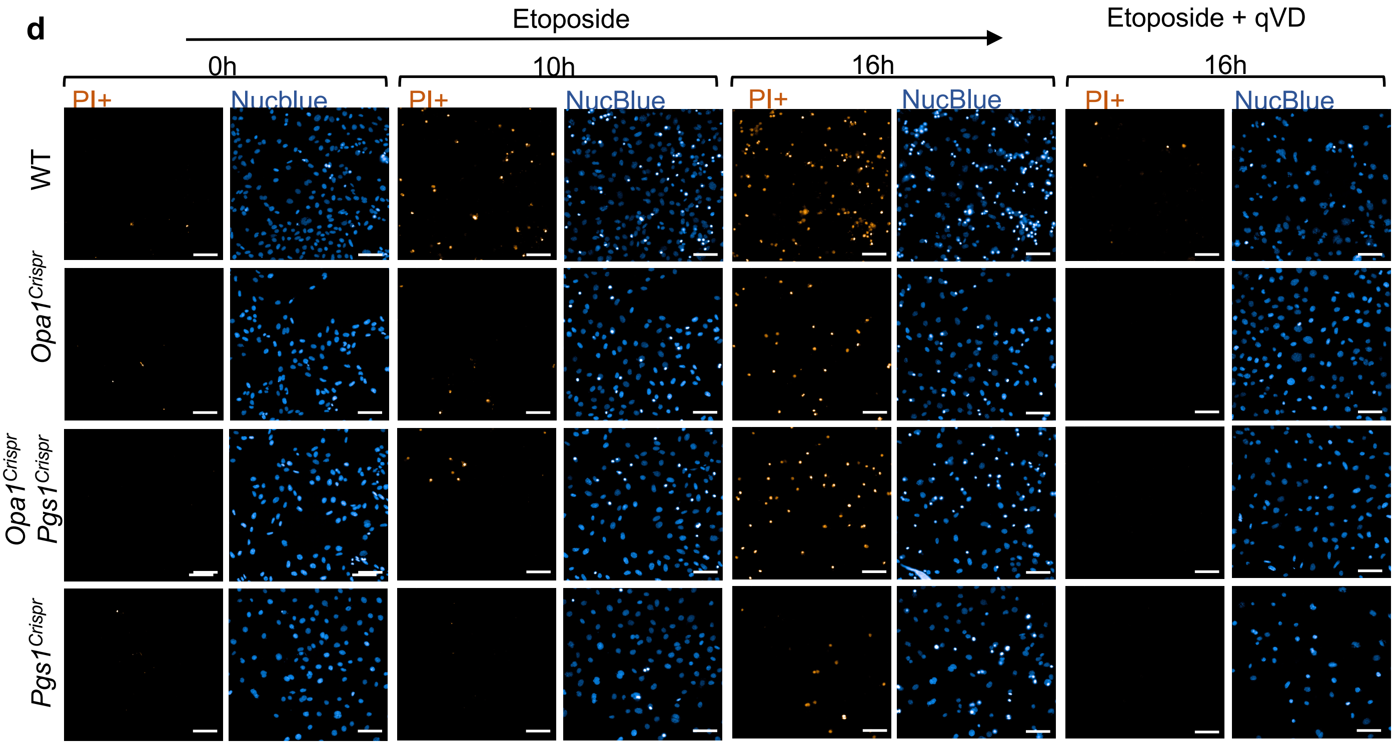

Appendix Figure S8: Kinetic cell death imaging in MEFs triggered by etoposide

(A, B) MEFs of the indicated genotypes were subjected to 16 µM etoposide in the presence or absence of the pan-caspase inhibitor qVD. Dead cells (PI+ nuclei, orange) and total cells (NucBlue, blue) were imaged every hour for 25 hours. PI+ nuclei number divided by the total nuclei number was then quantified over time. Representative confocal images of (B). Scale bar=100µm. Data represent mean ± SD of four independent experiments, 1816-3915 cells per cell line) One-way ANOVA; ****p < 0.0001.
