# Supplementary material for: High‐throughput screening identifies suppressors of mitochondrial fragmentation in OPA1 fibroblasts
Source: EMBO Mol Med. 2021 May 20;13(6):e13579. doi: 10.15252/emmm.202013579 (PMC8185549; doi:10.15252/emmm.202013579)
Supplement: Supplementary file 2 — Expanded View Figures PDF [file EMMM-13-e13579-s003.pdf]

Expanded View Figures

Figure EV1. Mitochondrial morphology supervised machine learning pipeline.

Mitochondrial morphology quantification workflow using PhenoLOGIC Harmony 4.9 supervised machine learning (Dataset EV1). Fluorescence imaging of cells (1) followed by nuclei (2) and cell segmentation (3) using cytoplasm defined by mitochondria. Cells on the border of the field of view are excluded from downstream analyses (4). Mitochondrial signal intensity is transformed into various texture parameters (5). (6) Supervised ML is trained using cells harboring mitochondria that are fragmented (*Opa1* siRNA, red), hypertubulated (*Dnm1l* siRNA, blue), or normal (NT siRNA, green). Debris cells (yellow) not included in the classification. (7) Class segregation represented by goodness of fit and then applied to unknown, non-trained images (8).

 © 2021 The Authors

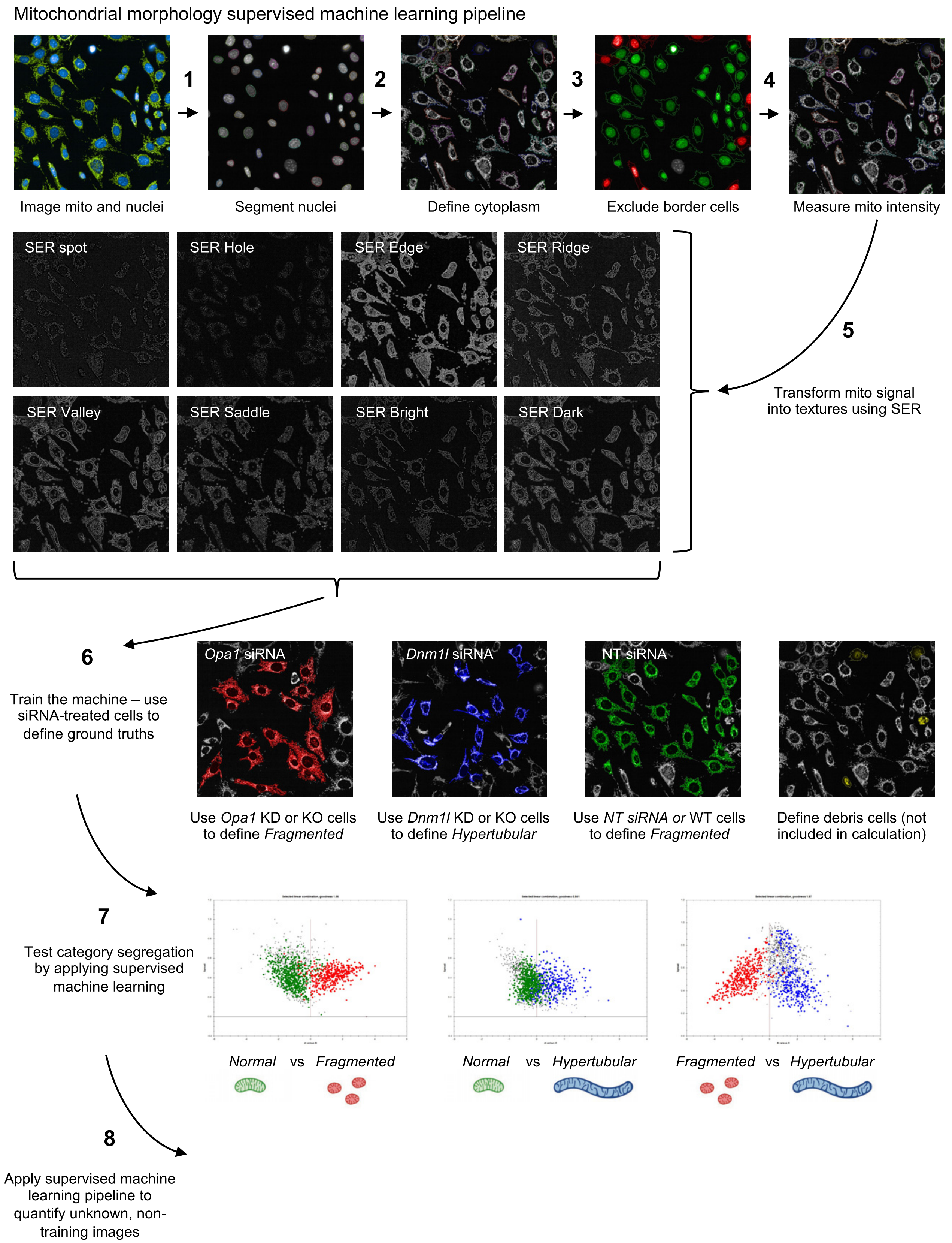
Mitochondrial morphology supervised machine learning pipeline
1
2
3
4
Image mito and nuclei
Segment nuclei
Define cytoplasm
Exclude border cells
Measure mito intensity
SER spot
SER Hole
SER Edge
SER Ridge
SER Valley
SER Saddle
SER Bright
SER Dark
5
Transform mito signal into textures using SER
6
Train the machine – use siRNA-treated cells to define ground truths
Opa1 siRNA
Dnm1l siRNA
NT siRNA
Use Opa1 KD or KO cells to define Fragmented
Use Dnm1l KD or KO cells to define Hypertubular
Use NT siRNA or WT cells to define Fragmented
Define debris cells (not included in calculation)
7
Test category segregation by applying supervised machine learning
Normal vs Fragmented
Normal vs Hypertubular
Fragmented vs Hypertubular
8
Apply supervised machine learning pipeline to quantify unknown, non-training images

Figure EV1.

Figure EV2. High-throughput screening identifies known and novel genetic modifiers of mitochondrial morphology in control and DOA+ patient-derived fibroblasts.

A, B Representative confocal images of candidate genes (siRNAs) able to fragment (B) or hypertubulate (A) mitochondrial morphology identified in *Mitome* screen of *control* (CTL-1) fibroblasts (Fig 2A–C) Scale bar = 50 μm.

C, D Representative confocal images of candidate genes (siRNAs) able to rescue (C) or hyperfragment (D) mitochondrial morphology identified in *Mitome* screen of $OPA1^{S545R}$ patient fibroblasts (Fig 2E and F) Scale bar = 50 μm.

E Violin plot representing % Hyperfragmented morphology of ground truth and *Mitome* siRNAs. The siRNA able to hyperfragment mitochondrial morphology in $OPA1^{S545R}$ patient fibroblasts were selected with a univariate 3-components statistical model programmed in R using ground truths for morphology in Fig 2E. The defined threshold for positive hits was 72.9% (dotted line) and identified 27 candidate genes (Dataset EV3) from the same experiment as shown in Fig 2E.

F, G (F) Representative confocal images and (G) mitochondrial morphology quantification of control (CTL-1) fibroblasts and $OPA1^{S545R}$ patient fibroblasts treated with indicated siRNAs for 72 h. Scale bar=50μm. Supervised ML training performed on cells with fragmented (*OPA1* siRNA), normal (non-targeting NT siRNA), and hypertubulated (*DNM1L* siRNA) mitochondria. Data represent mean ± SD of measurement of 419–1,783 cells performed once.

H Functional classification of 91candidate genes that rescued mitochondrial fragmentation in $OPA1^{S545R}$ fibroblasts upon depletion (Dataset EV3).

© 2021 The Authors

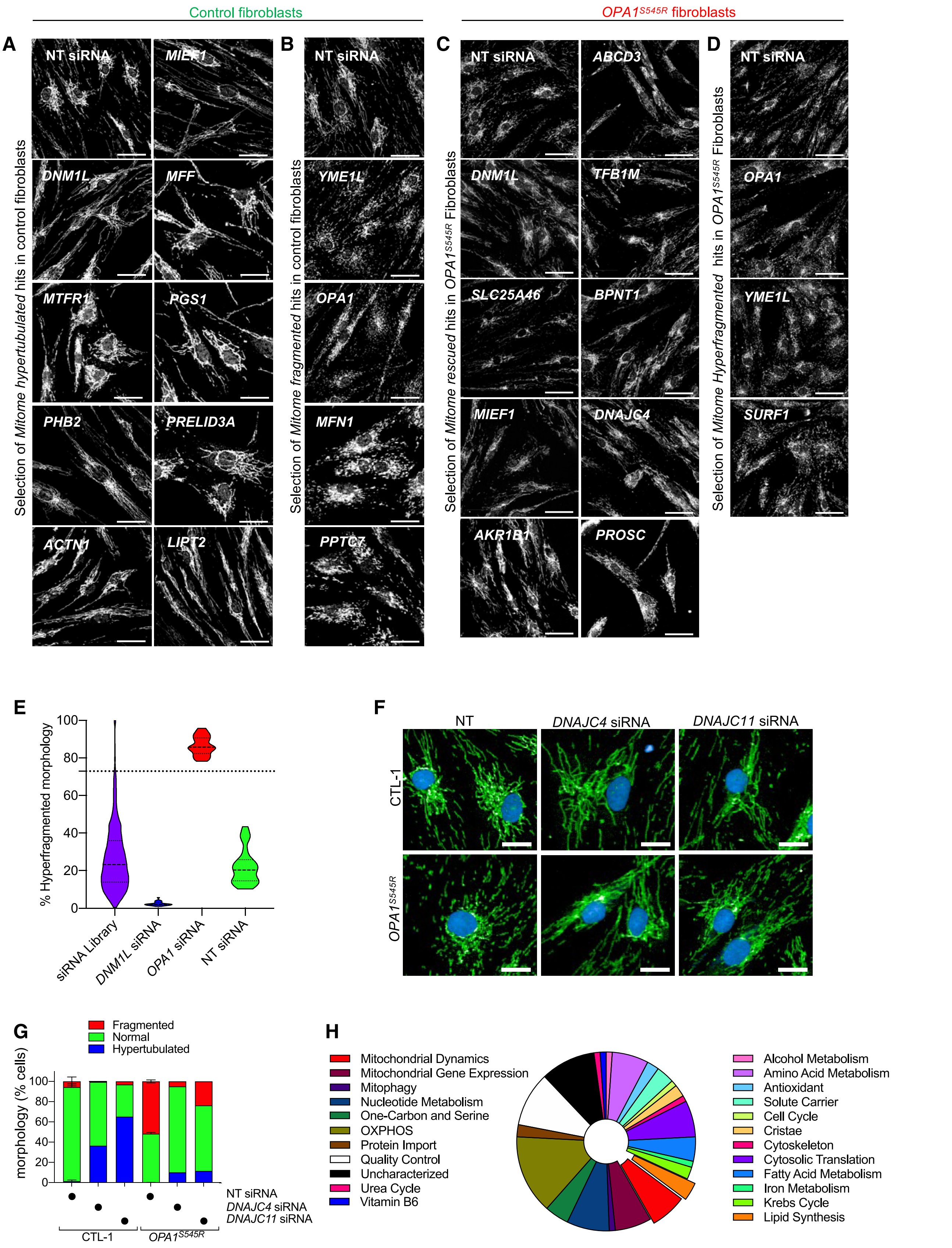
Control fibroblasts
OPA1S545R fibroblasts
A
B
C
D
NT siRNA
MIEF1
DNM1L
MFF
MTFR1
PGS1
PHB2
PRELID3A
ACTN1
LIPT2
Selection of Mitome hypertubulated hits in control fibroblasts
YME1L
OPA1
MFN1
PPTC7
Selection of Mitome fragmented hits in control fibroblasts
ABCD3
TFB1M
SLC25A46
BPNT1
DNAJC4
AKR1B1
PROSC
Selection of Mitome rescued hits in OPA1S545R Fibroblasts
SURF1
Selection of Mitome Hyperfragmented hits in OPA1S545R Fibroblasts
E
% Hyperfragmented morphology
siRNA Library
DNM1L siRNA
OPA1 siRNA
NT siRNA
F
NT
DNAJC4 siRNA
DNAJC11 siRNA
CTL-1
OPA1S545R
G
Fragmented
Normal
Hypertubulated
morphology (% cells)
NT siRNA
DNAJC4 siRNA
DNAJC11 siRNA
CTL-1
H
Mitochondrial Dynamics
Mitochondrial Gene Expression
Mitophagy
Nucleotide Metabolism
One-Carbon and Serine
OXPHOS
Protein Import
Quality Control
Uncharacterized
Urea Cycle
Vitamin B6
Alcohol Metabolism
Amino Acid Metabolism
Antioxidant
Solute Carrier
Cell Cycle
Cristae
Cytoskeleton
Cytosolic Translation
Fatty Acid Metabolism
Iron Metabolism
Krebs Cycle
Lipid Synthesis

Figure EV2.

Figure EV3. PGS1 depletion rescues mitochondrial fragmentation in OPA1-deficient human and mouse fibroblasts.

A *Opa1* splice isoforms in MEFs. Proteolytic cleavage sites for MPP, OMA1, and YME1L are indicated. TM, transmembrane domain.

B Equal amounts of protein extracted from WT and *Opa1*^{KO} MEFs were separated by SDS–PAGE, immunoblotted with indicated antibodies. WT and *Opa1*^{Crispr} immunoblots from Fig 3G are cropped and included for reference.

C Quantitative RT–PCR (qRT–PCR) measurement of *Opa1* and *Pgs1* expression in indicated *Opa1*^{Crispr} MEFs complemented with pLenti-Opa1 and *Opa1*^{Crispr}*Pgs1*^{Crispr} MEFs complemented with pLenti-Pgs1 relative to WT control. Data represent mean ± SD of two independent experiments, unpaired *t*-test; $^{**}P < 0.01$, $^{****}P < 0.0001$, ns; not significant.

D (left) quantitative RT–PCR (qRT–PCR) measurement of normalized *Opa1* expression (relative to *Gapdh*) WT MEFs treated with *Opa1* siRNA for 72 h relative to NT (non-targeting) control. Data represent mean ± SD of 4 independent experiments, unpaired *t*-test; $^{***}P < 0.001$. (right) Equal amounts of protein extracted from WT MEFs treated with indicated siRNAs for 72 h were separated by SDS–PAGE, immunoblotted for OPA1.

E Representative confocal images of WT, *Opa1*^{Crispr}, and *Opa1*^{Crispr} MEFs complemented with pLenti-Opa1-Myc. Mitochondria (mitoYFP, green) and nuclei (DAPI, blue) and Opa1-Myc (anti-Myc, red). Scale bar = 10 μm.

F Mitochondrial morphology quantification of (E) using WT MEFs treated with *Opa1* siRNA (fragmented), NT siRNA (normal), or *Dnm1l* siRNA (hypertubulated) ground truth training sets. Data represent mean ± SD of six independent experiments (1,693–3,200 cells per cell line), One-way ANOVA (% fragmented); $^{***}P < 0.001$, ns; not significant.

G Equal amounts of protein extracted from MEFs were separated by SDS–PAGE, immunoblotted with indicated antibodies. Horizontal line denotes separate membranes.

H Mitochondrial membrane potential measured in WT, *Opa1*^{Crispr}, *Opa1*^{Crispr} + pLenti-Opa1-Myc MEFs labeled with TMRE and analyzed by flow cytometry. TMRE/mitoYFP was used to normalize membrane potential. Data represent mean ± SD of three independent experiments (> 10,000 cells/sample), One-way ANOVA.

© 2021 The Authors

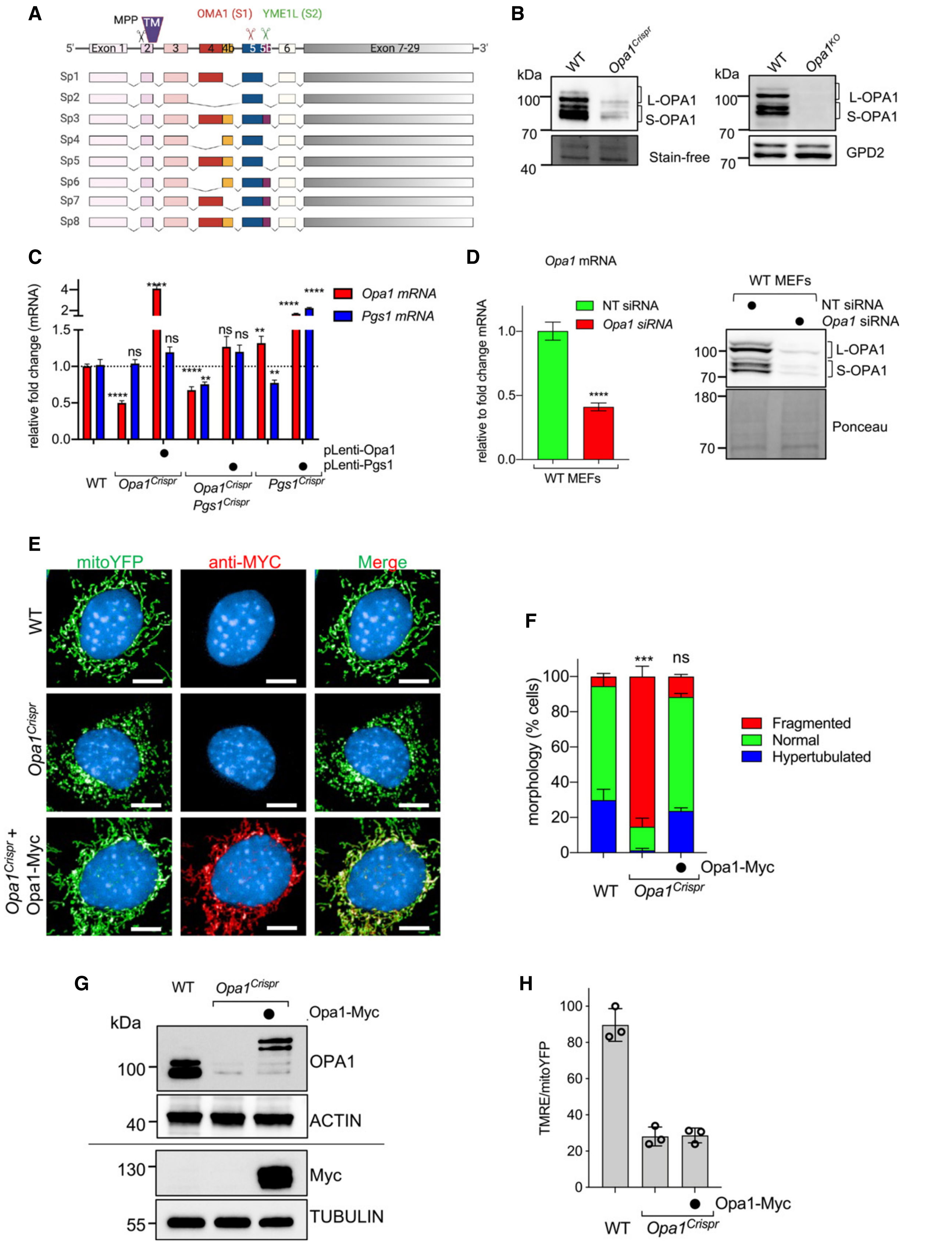
A
MPP
TM
OMA1 (S1)
YME1L (S2)
Exon 1
Exon 7-29
Sp1
Sp2
Sp3
Sp4
Sp5
Sp6
Sp7
Sp8
B
kDa
WT
Opa1Crispr
L-OPA1
S-OPA1
Stain-free
Opa1KO
GPD2
C
relative fold change (mRNA)
Opa1 mRNA
Pgs1 mRNA
pLenti-Opa1
pLenti-Pgs1
Pgs1Crispr
D
Opa1 mRNA
NT siRNA
Opa1 siRNA
relative to fold change mRNA
WT MEFs
Ponceau
E
mitoYFP
anti-MYC
Merge
Opa1Crispr + Opa1-Myc
F
morphology (% cells)
Fragmented
Normal
Hypertubulated
Opa1-Myc
G
OPA1
ACTIN
Myc
TUBULIN
H
TMRE/mitoYFP

Figure EV3.

Figure EV4. PGS1 depletion rescues mitochondrial fragmentation by inhibiting mitochondrial fission independently of DRP1 recruitment and OPA1 processing.

A, B (A) Equal amounts of protein extracted from WT and mutant MEFs stably expressing pLenti-Opa1 or pLenti-Pgs1 where indicated were separated by SDS–PAGE, immunoblotted with indicated antibodies and quantified by densitometry (B) relative to tubulin. Data represent mean ± SD of four independent experiments.

C Equal amounts of protein extracted from WT MEFs treated with 20 μM CCCP (30 min) or 16 μM 4Br-A23187 (18 h) were separated by SDS–PAGE, immunoblotted with indicated antibodies. Tubulin was used as loading control.

D Representative confocal images of MEFs knocked in for mTurquoise2-Dnm1l by Crispr/Cas9 genome editing in WT (WT^{Drp1KI}) and $Opa1^{Crispr}$ ($Opa1^{Crispr\text{-}DrpKI}$) MEFs treated with non-targeting (NT) or *Pgs1* siRNA for 72 h. DRP1 (mTurquoise2, purple), mitochondria (mitoYFP, green). Scale bar = 20 μm.

E Bar graph representation of DRP1 localized to mitochondria (blue) vs cytosol (green). Data represent mean ± SD of five replicates (193–1,062 cells per cell line), One-way ANOVA.

Source data are available online for this figure.

© 2021 The Authors

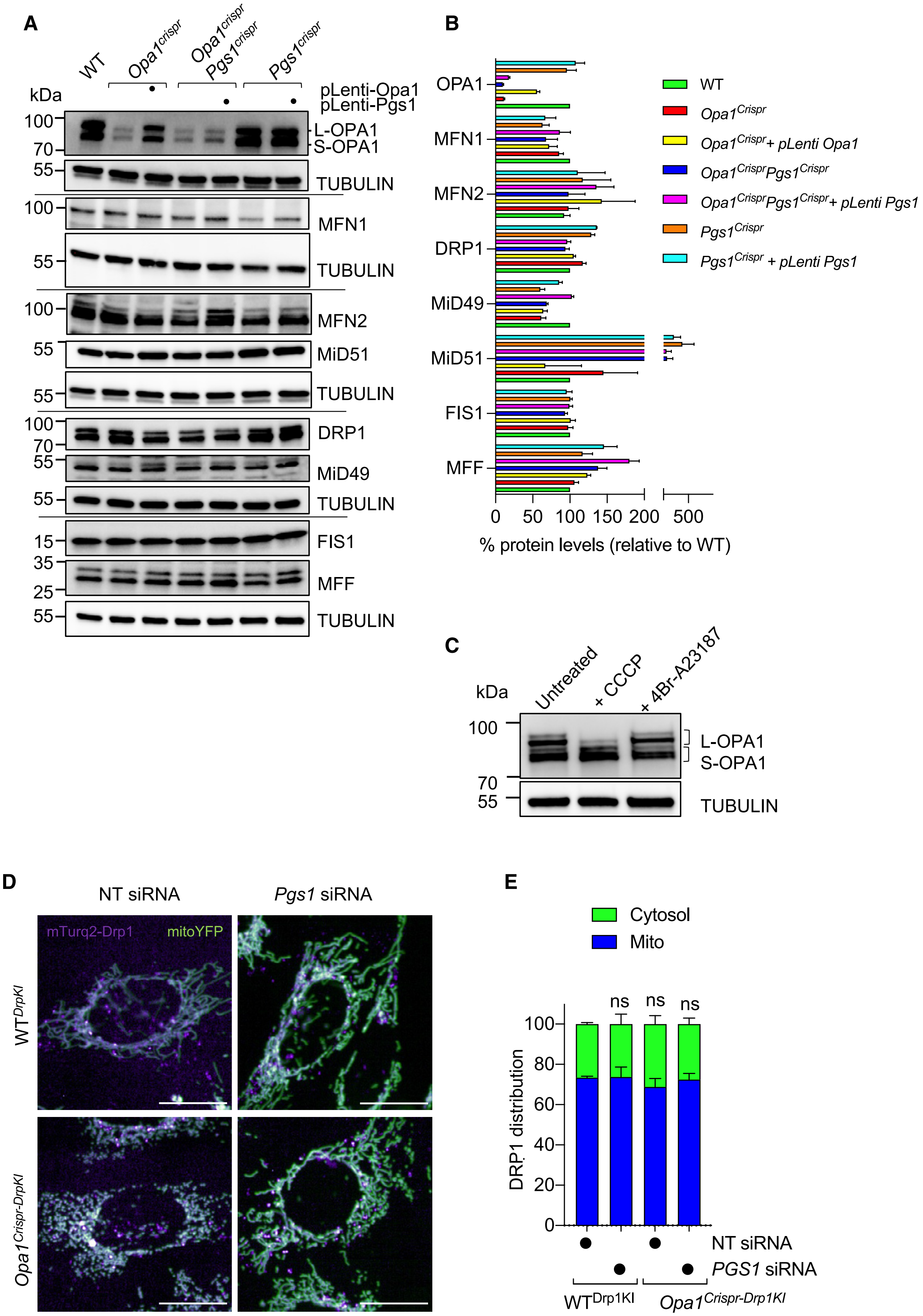

A
WT
Opa1crispr
Opa1crispr Pgs1crispr
Pgs1crispr
pLenti-Opa1
pLenti-Pgs1
kDa
L-OPA1
S-OPA1
TUBULIN
MFN1
MFN2
MiD51
DRP1
MiD49
FIS1
MFF
B
OPA1
MFN1
MFN2
DRP1
MiD49
MiD51
FIS1
MFF
WT
Opa1Crispr
Opa1Crispr+ pLenti Opa1
Opa1CrisprPgs1Crispr
Opa1CrisprPgs1Crispr+ pLenti Pgs1
Pgs1Crispr
Pgs1Crispr + pLenti Pgs1
% protein levels (relative to WT)
C
Untreated
+ CCCP
+ 4Br-A23187
L-OPA1
S-OPA1
TUBULIN
D
NT siRNA
Pgs1 siRNA
mTurq2-Drp1
mitoYFP
WTDrpKI
Opa1Crispr-DrpKI
E
Cytosol
Mito
DRP1 distribution
ns
NT siRNA
PGS1 siRNA
WTDrp1KI
Opa1Crispr-Drp1KI

Figure EV4.

Figure EV5. Cardiolipin remodeling in OPA1 and PGS1-deficient fibroblasts.

A Representative confocal micrographs of MEFs WT and *Opa1*^{KO} MEFs treated with indicated siRNAs for 72 h. Mitochondria (anti-TOMM40, green) and nuclei (DAPI, blue). Scale bar = 10 μm.

B Mitochondrial morphology quantification of (a) using WT MEFs treated with *Opa1* siRNA (fragmented), NT siRNA (normal), or *Dnm1l* siRNA (hypertubulated) training sets. Data represent mean ± SD of two independent experiments, (185–2,689 cells per cell line), One-way ANOVA (% fragmented); ****$P < 0.0001$.

C Representative confocal images of wild-type (WT) and *Yme1l*$^{-/-}$ MEFs treated with indicated siRNAs for 72 h. Live imaging of mitochondria labeled with MitoTracker Deep Red (MTDR, green), TMRE (pink), and NucBlue (Nuclei, blue). Scale bar = 10 μm.

D Supervised ML mitochondrial morphology quantification of (c) using WT MEFs treated with *Opa1* siRNA (fragmented), NT siRNA (normal), or *Dnm1l* siRNA (hypertubulated) training sets. *Opa1*^{KO} MEFs (from Fig EV3B) are represented for comparison.

E Quantification of mitochondrial membrane potential (TMRE/MTDR) of cells imaged in (C). Number of analyzed cells inset. Data represent mean ± SD of one independent experiments, One-way ANOVA; ***$P < 0.001$, ****$P < 0.0001$.

 © 2021 The Authors

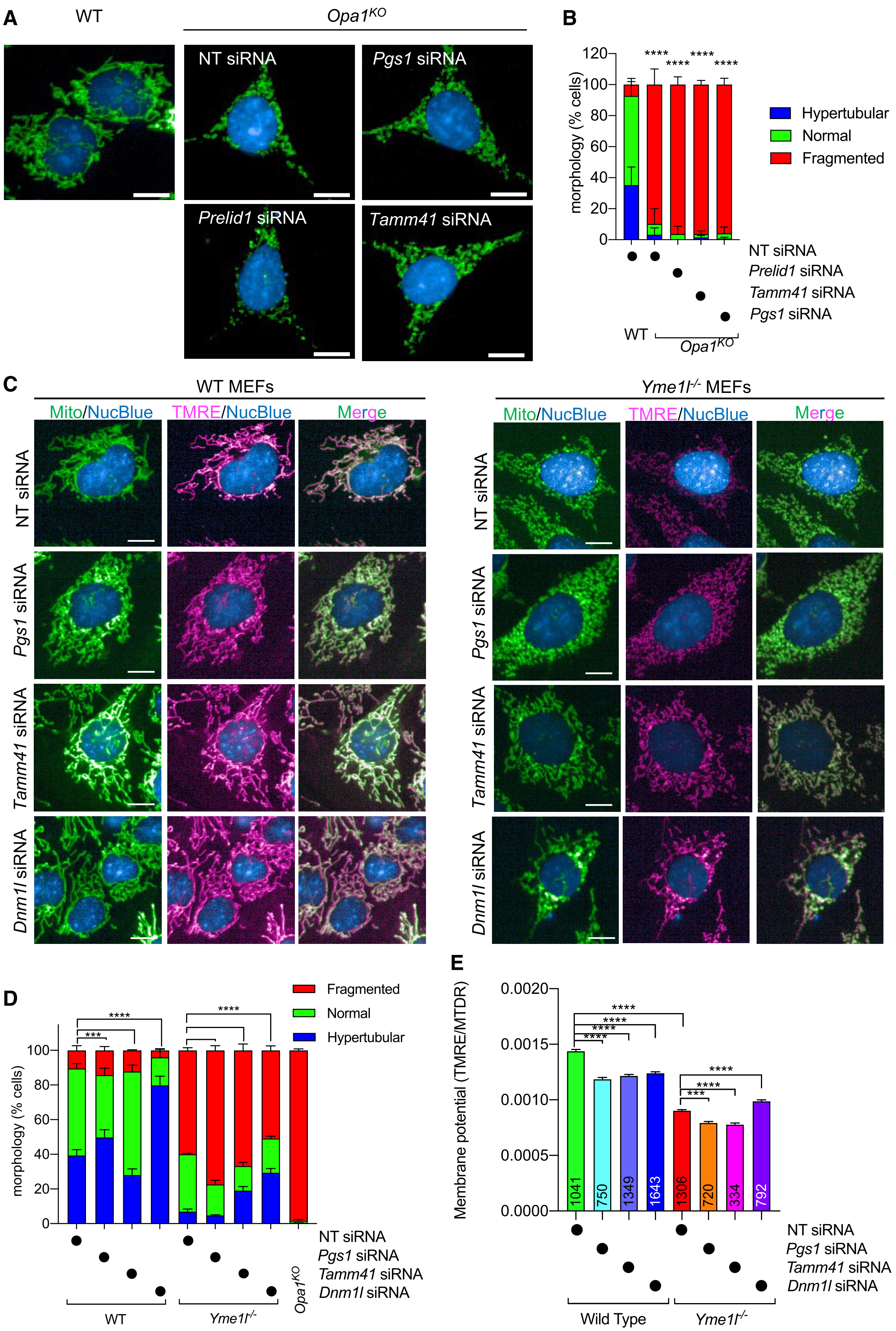
A
WT
Opa1KO
NT siRNA
Pgs1 siRNA
Prelid1 siRNA
Tamm41 siRNA
B
morphology (% cells)
Hypertubular
Normal
Fragmented
NT siRNA
Prelid1 siRNA
Tamm41 siRNA
Pgs1 siRNA
WT
Opa1KO
C
WT MEFs
Yme1l-/- MEFs
Mito/NucBlue
TMRE/NucBlue
Merge
NT siRNA
Pgs1 siRNA
Tamm41 siRNA
Dnm1l siRNA
D
morphology (% cells)
Fragmented
Normal
Hypertubular
NT siRNA
Pgs1 siRNA
Tamm41 siRNA
Dnm1l siRNA
WT
Yme1l-/-
Opa1KO
E
Membrane potential (TMRE/MTDR)
1041
750
1349
1643
1306
720
334
792
NT siRNA
Pgs1 siRNA
Tamm41 siRNA
Dnm1l siRNA
Wild Type
Yme1l-/-

Figure EV5.

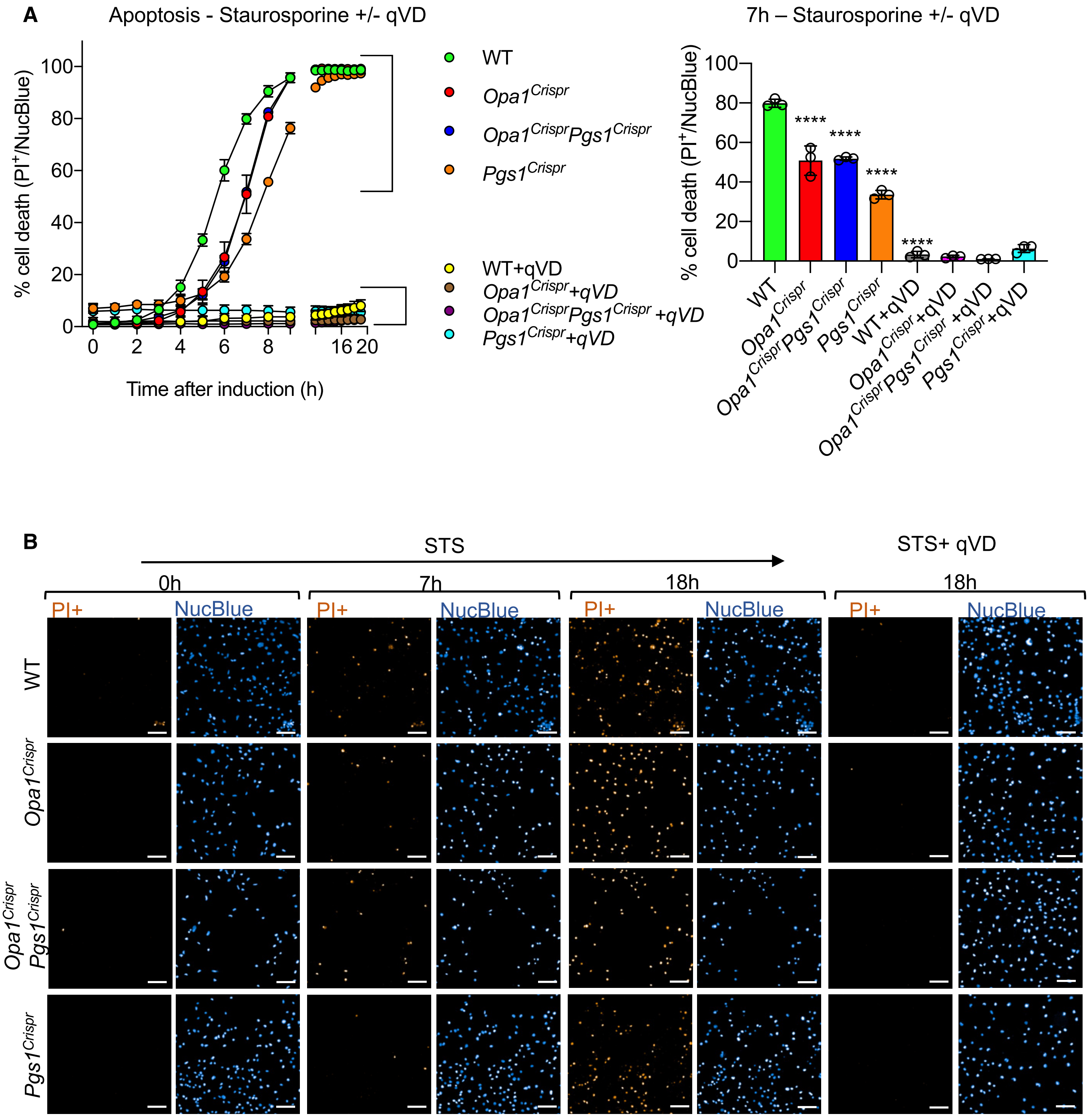

Figure EV6. PGS1 depletion does not restore apoptotic phenotype of OPA1-deficient MEFs.

A, B (A) MEFs of the indicated genotypes were subjected to 0.5 μM staurosporine in the presence or absence of the pan-caspase inhibitor qVD. Dead cells (PI^+ nuclei, orange) and total cells (NucBlue, blue) were imaged every hour for 25 h. PI^+ nuclei number divided by the total nuclei number was then quantified over time. (B) Representative confocal images of (A). Scale bar = 100 μm. Data represent mean ± SD of three independent experiments (1,923–4,703 cells per cell line) One-way ANOVA; ****$P < 0.0001$.

 © 2021 The Authors

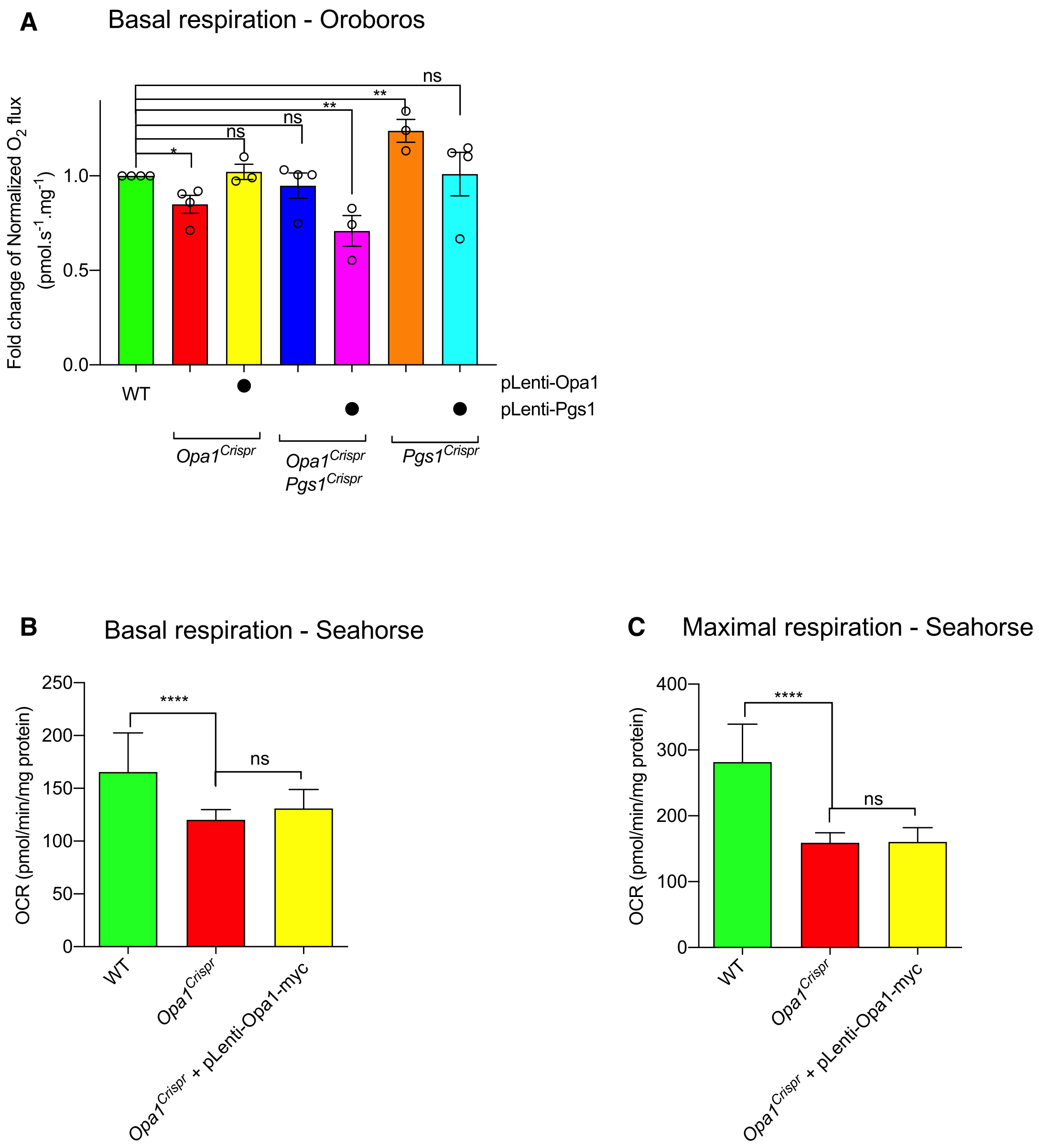

Figure EV7. OPA1-Myc does not complement respiration defect in OPA1-deficient MEFs.

A Basal oxygen consumption (JO_2 flux) measured by O2k high-resolution respirometry (Oroboros) in WT, *Opa1*^{Crispr}, *Opa1*^{Crispr} + pLenti-Opa1, *Opa1*^{Crispr}*Pgs1*^{Crispr}, *Opa1*^{Crispr}*Pgs1*^{Crispr} + pLenti-Pgs1, *Pgs1*^{Crispr} and *Pgs1*^{Crispr} MEFs + pLenti-Pgs1. Measurements were made in pairwise fashion compared with WT MEFs. O_2 flux normalized to protein concentration. Data represent mean ± SD of at least three independent experiments, unpaired *t*-test; *$P < 0.05$, **$P < 0.01$, ns, not significant.

B, C Mitochondrial respiration measured in adherent MEFs of the indicated genotypes using Seahorse FluxAnalyzer. Oxygen consumption rate (OCR) normalized to protein concentration. Following basal respiration, cells were treated sequentially with 1 μM Oligomycin (Omy), 2 μM CCCP (maximal). Bar graphs of representing basal (B) and maximal (C) respiration. Data represent mean ± SEM of 22–24 parallel OCR measurement, One-way ANOVA; ****$P < 0.0001$, ns, not significant.

© 2021 The Authors
