# Supplementary material for: High‐throughput screening identifies suppressors of mitochondrial fragmentation in OPA1 fibroblasts
Source: EMBO Mol Med. 2021 May 20;13(6):e13579. doi: 10.15252/emmm.202013579 (PMC8185549; doi:10.15252/emmm.202013579)
Supplement: Supplementary file 11 — Movie EV3 [file EMMM-13-e13579-s001.zip › Movie EV3.docx]

**Movie EV3:** FRAP fusion assay in mitoYFP WT (Movie EV1), *Opa1^Crispr^* (Move EV2) and *Opa1^Crispr^Pgs1^Crispr^* (Movie EV3) MEFs imaged in Figure 4E every 200ms. Movies represented at 5 frames per second.
