# Supplementary material for: High‐throughput screening identifies suppressors of mitochondrial fragmentation in OPA1 fibroblasts
Source: EMBO Mol Med. 2021 May 20;13(6):e13579. doi: 10.15252/emmm.202013579 (PMC8185549; doi:10.15252/emmm.202013579)
Supplement: Supplementary file 12 — Source Data for Expanded View/Appendix [file EMMM-13-e13579-s013.zip › EMM-2020-13579-V3-Appendix_Figure_S1D_Source_Data-sd.pdf]

Appendix Figure 1

S1D: OPA1 protein quantification relative to Ponceau stain.					
	DOA+-derived fibroblasts		n=1	n=2	n=3
		CTL-1	84.5379627	90.7399798	83.5449925
		CTL-3	115.462037	96.526833	102.01084
		OPA1 R445H	118.797351	121.724054	124.520906
		OPA1 S545R	82.0511548	75.8364744	77.5572624
		OPA1 I432X	114.468436	212.105958	159.000316
		OPA1 c.2356-1G>T	79.8171453	100.908459	77.6415854
		OPA1 Q297X	32.2192767	50.7322935	42.394682
		CTL-2		112.733187	114.444168

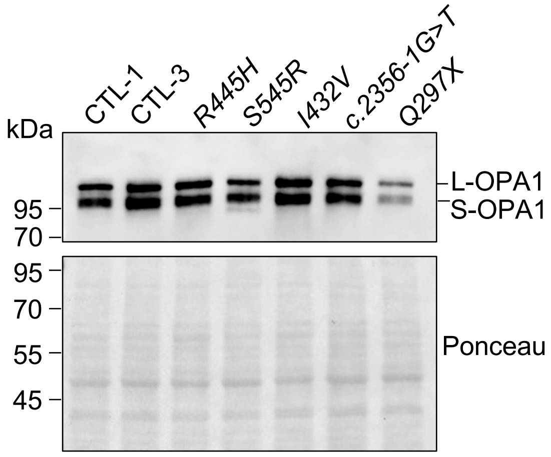

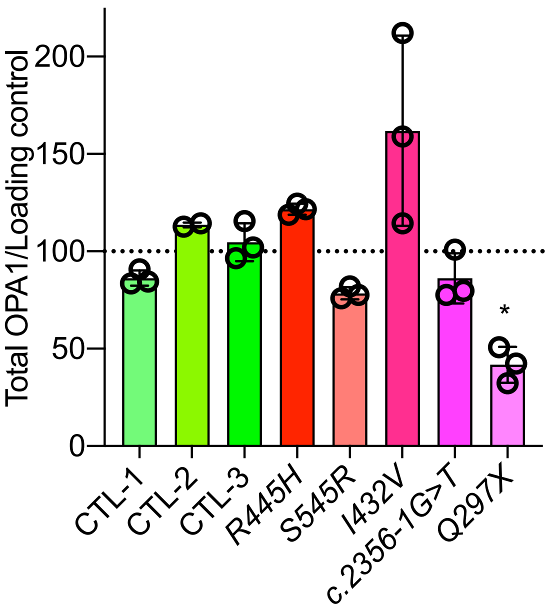

Figure S1

Source Data Appendix Figure S1D

EC249

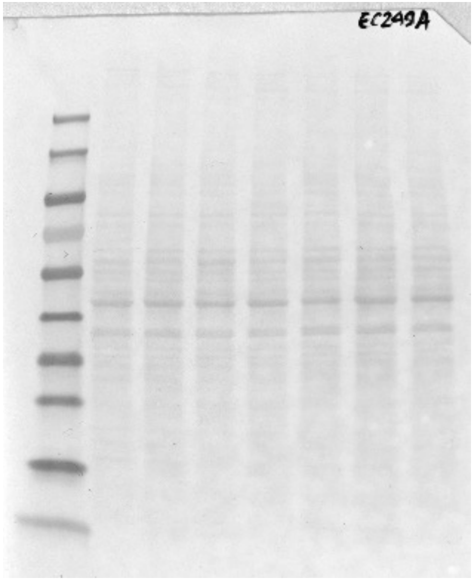

Loading

1st antibody

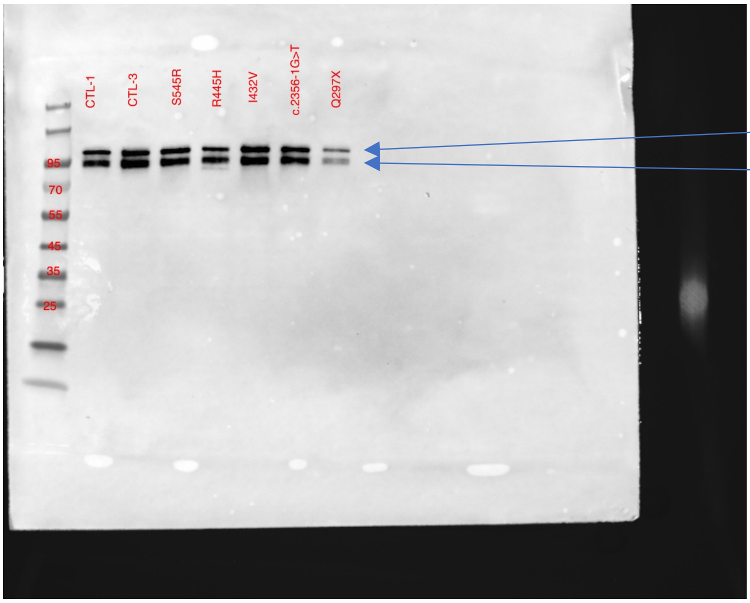

L-OPA1

S-OPA1

EC434

Loading

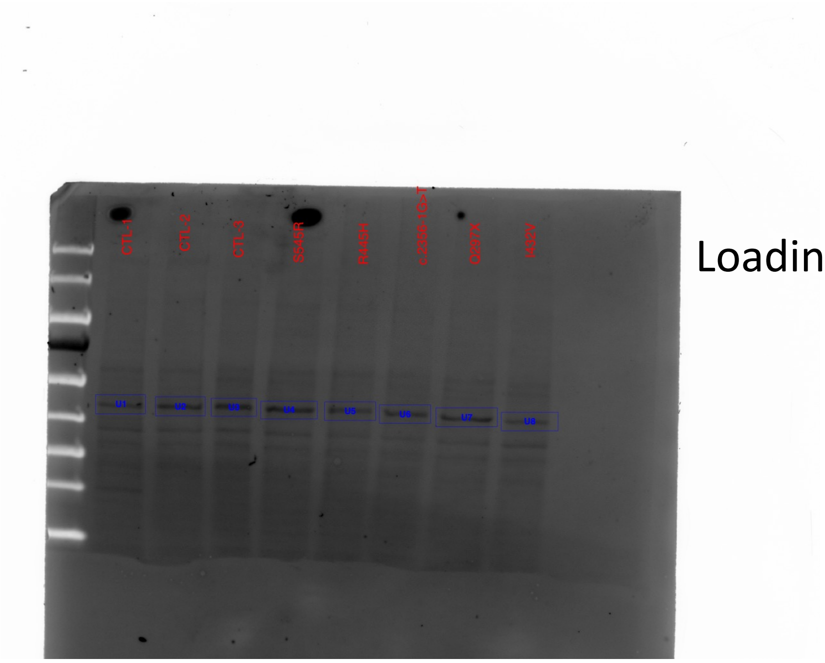

1st antibody

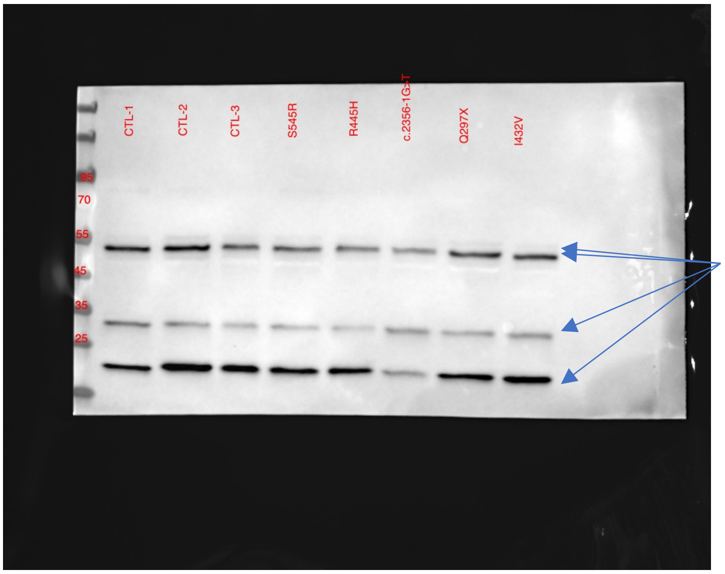

OXPHOS

2nd antibody

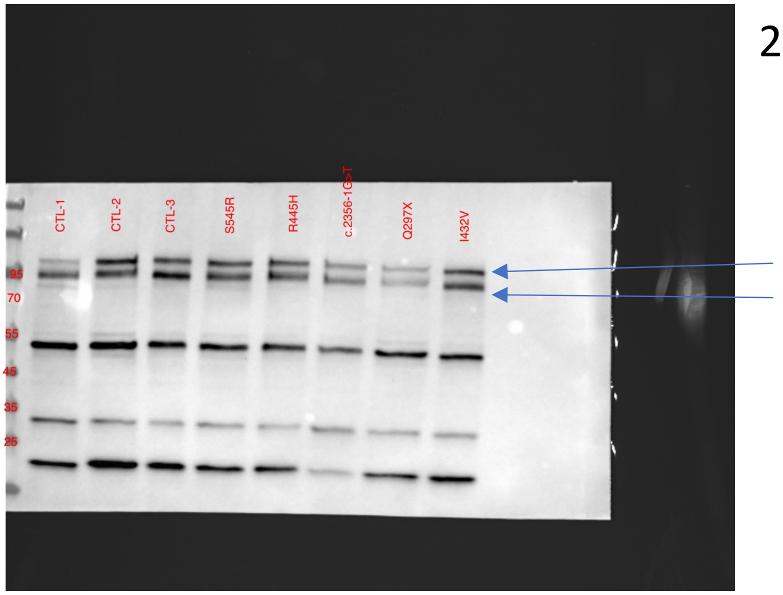

L-OPA1

S-OPA1

EC434

Loading

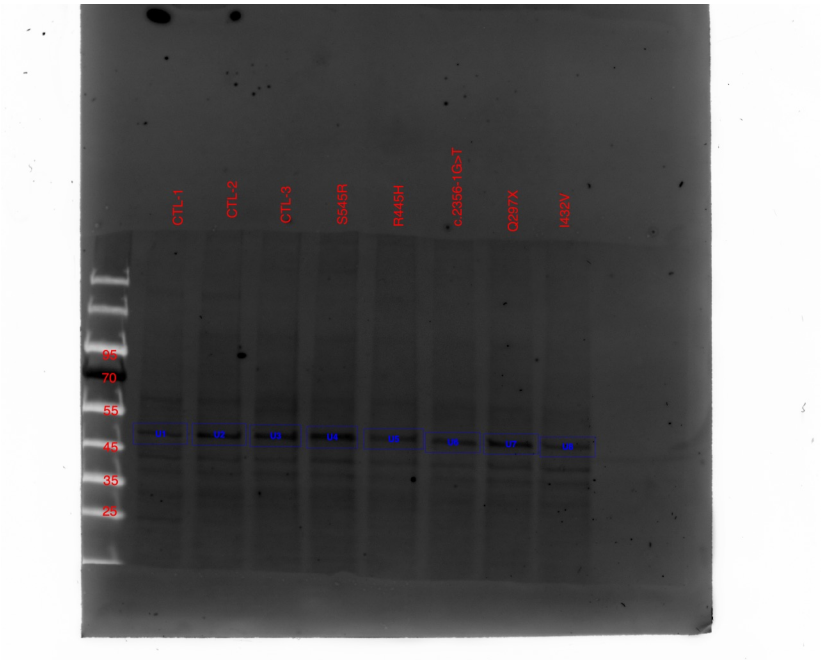

1st antibody

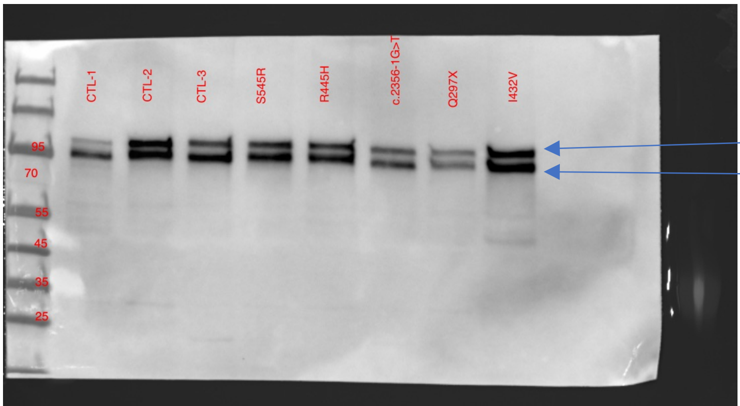

L-OPA1

S-OPA1

2nd antibody

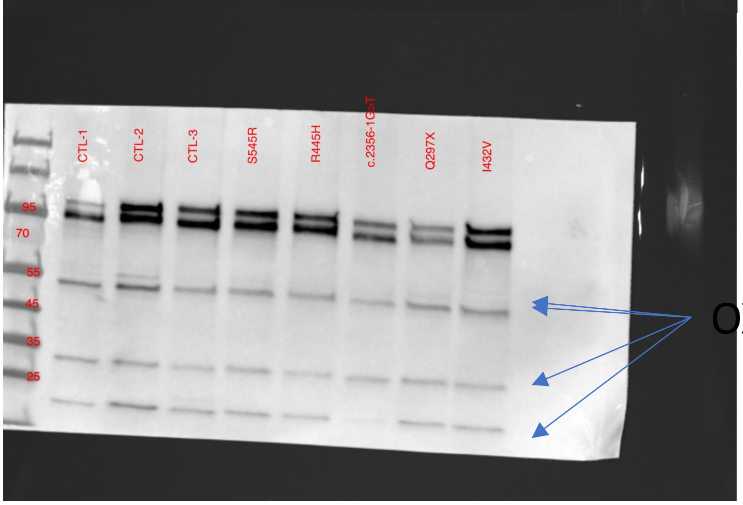

OXPHOS
