# Supplementary figures and images for: High‐throughput screening identifies suppressors of mitochondrial fragmentation in OPA1 fibroblasts
Source: EMBO Mol Med. 2021 May 20;13(6):e13579. doi: 10.15252/emmm.202013579 (PMC8185549; doi:10.15252/emmm.202013579)

Figure EV4a

Figure EV4\_Source data

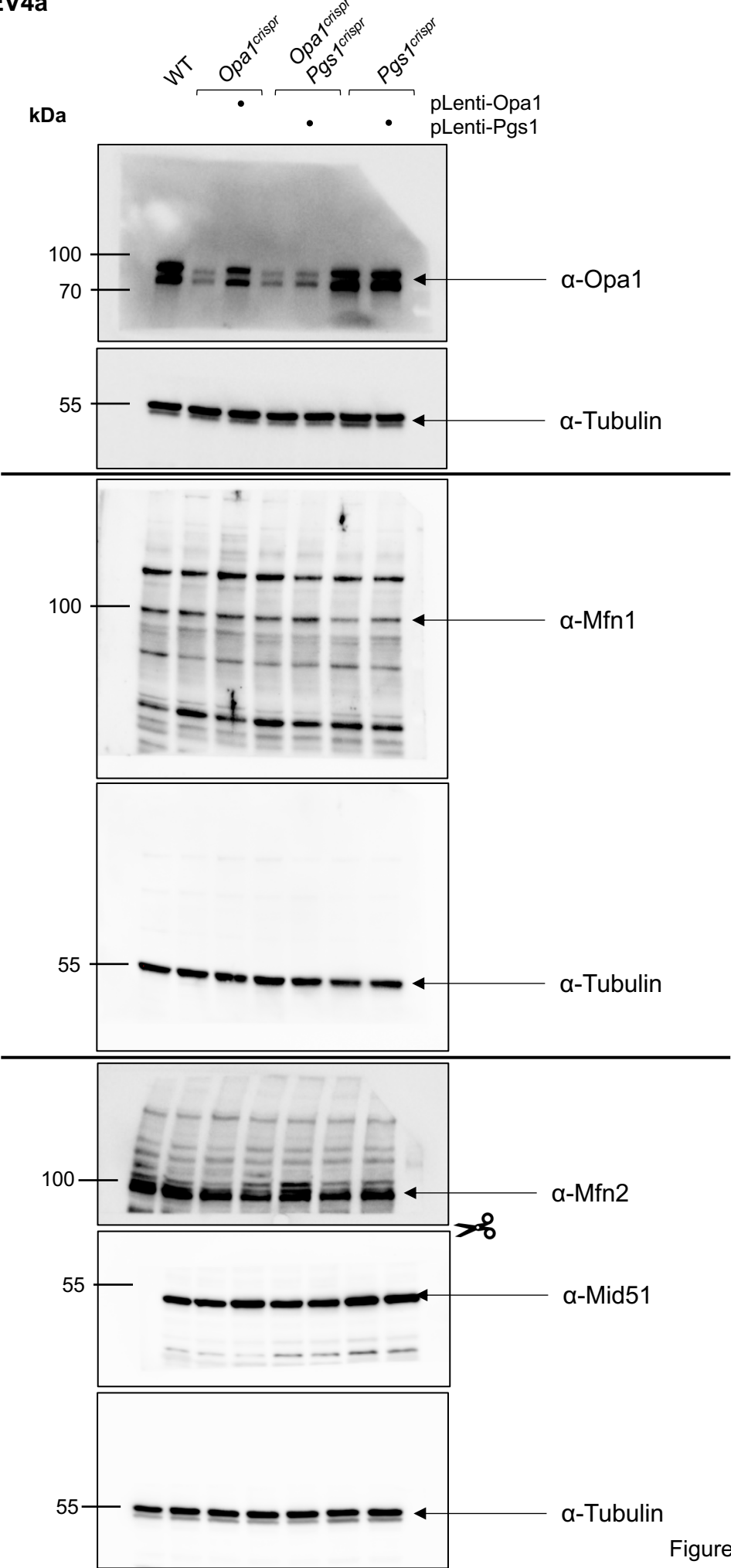

Figure EV4a cont.

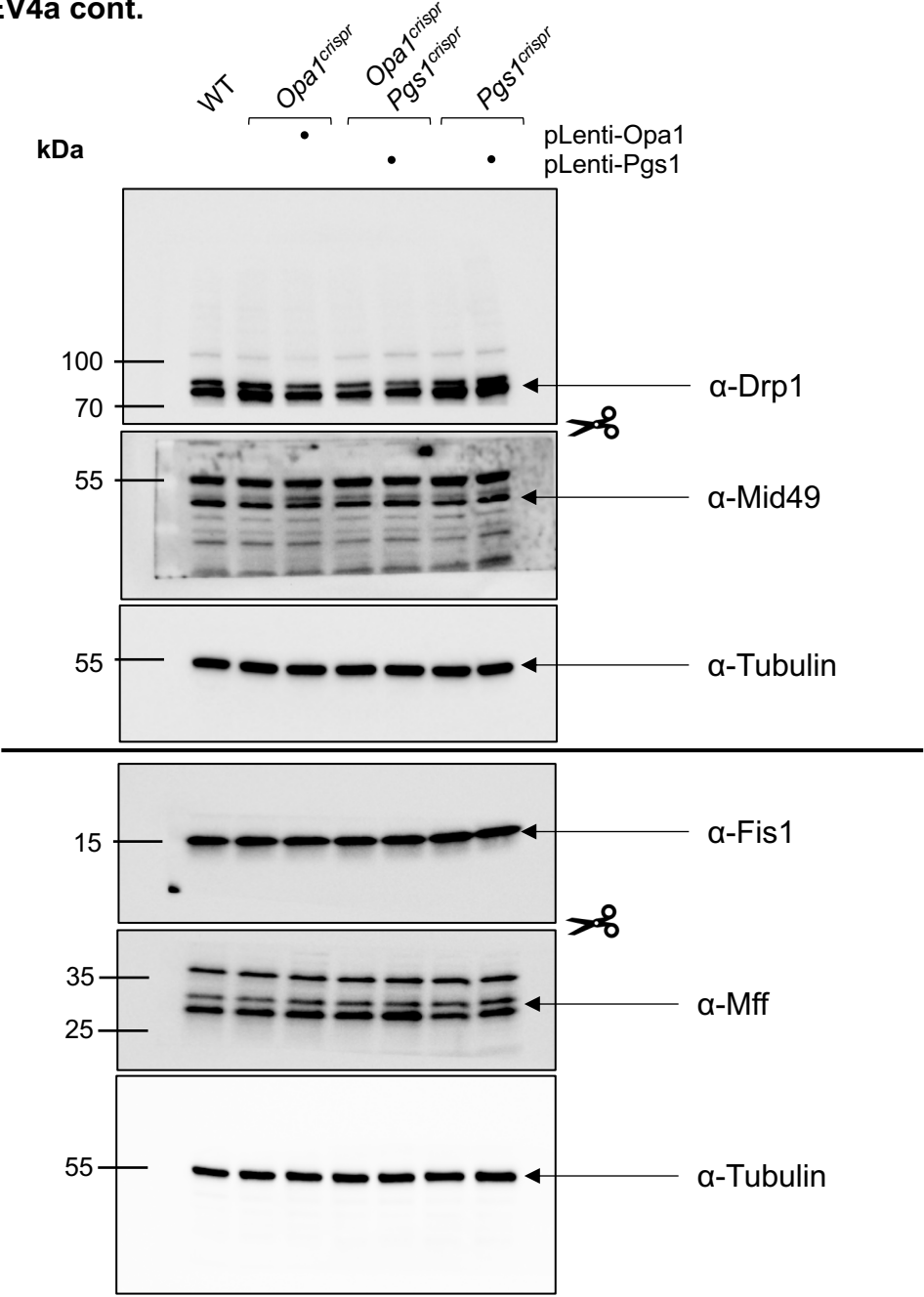

Figure EV4c

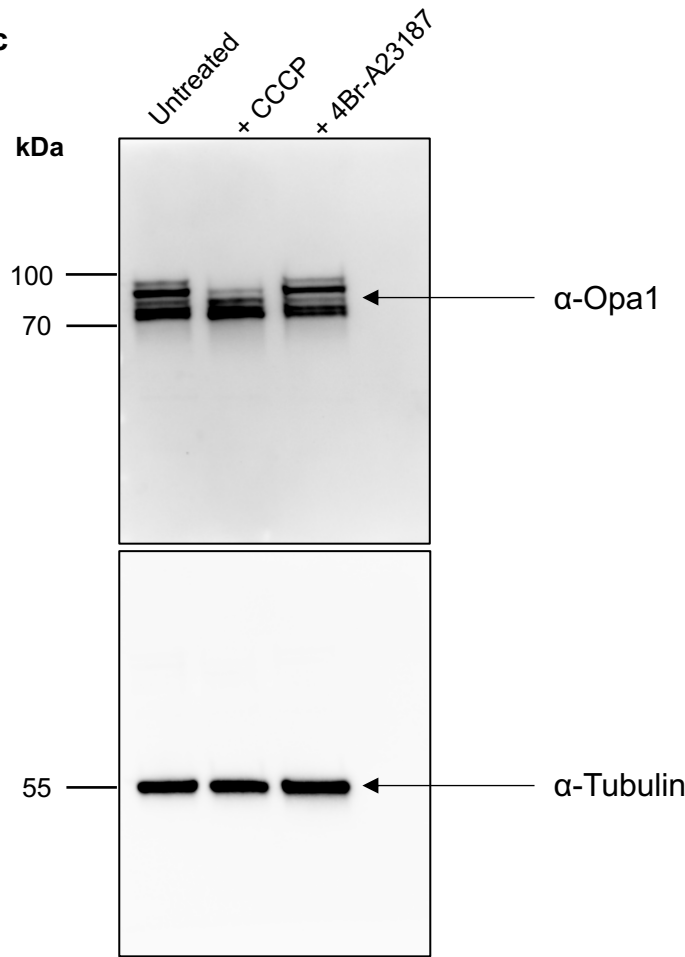

Figure EV4g

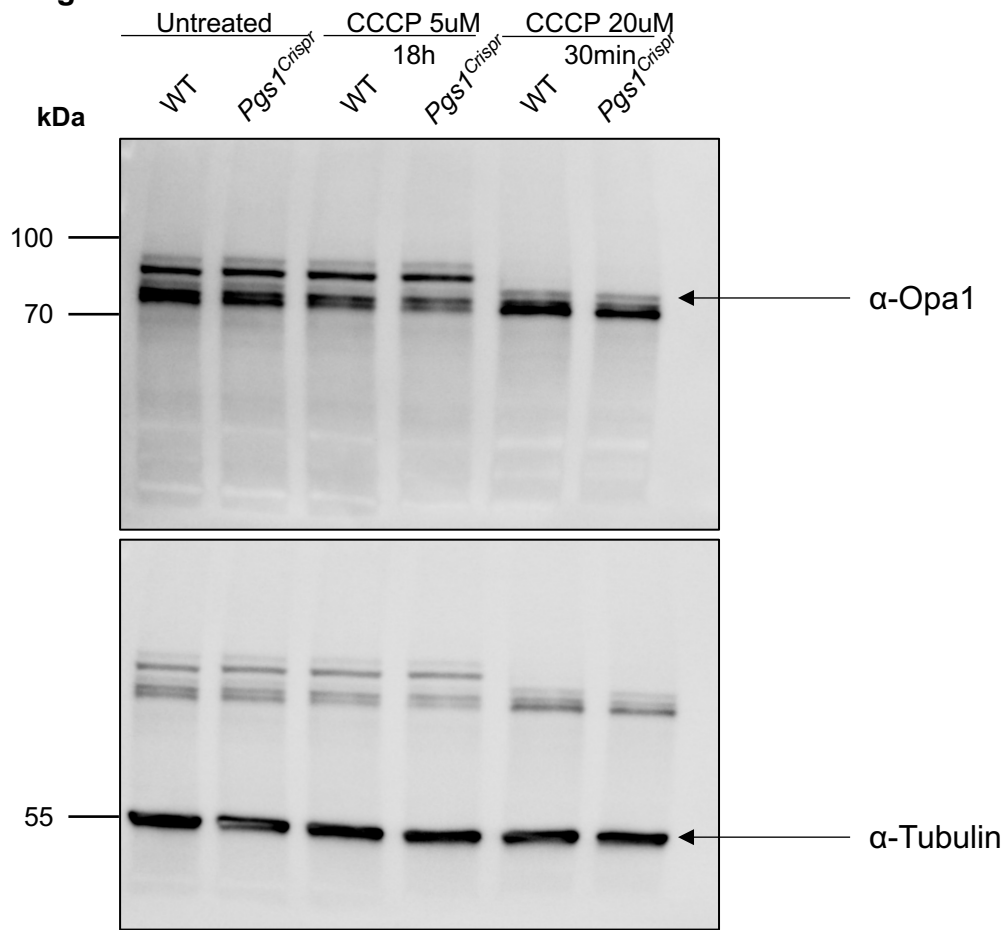

Supplement: Supplementary file 12 — Source Data for Expanded View/Appendix [file EMMM-13-e13579-s013.zip › EMM-2020-13579-V3-Figure_EV4_Source_Data-sd.pdf]

Figure 1B

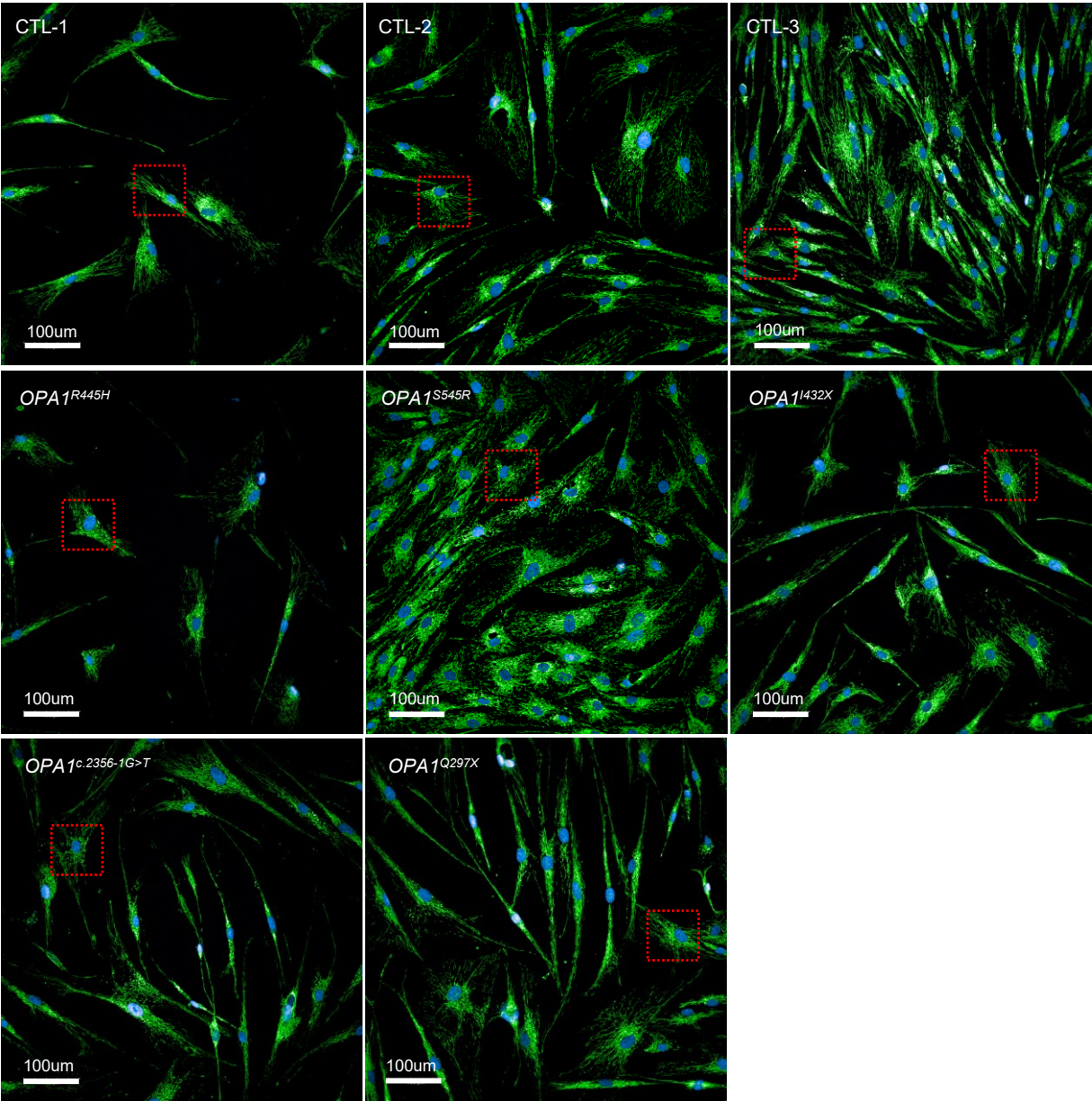

Figure 1D

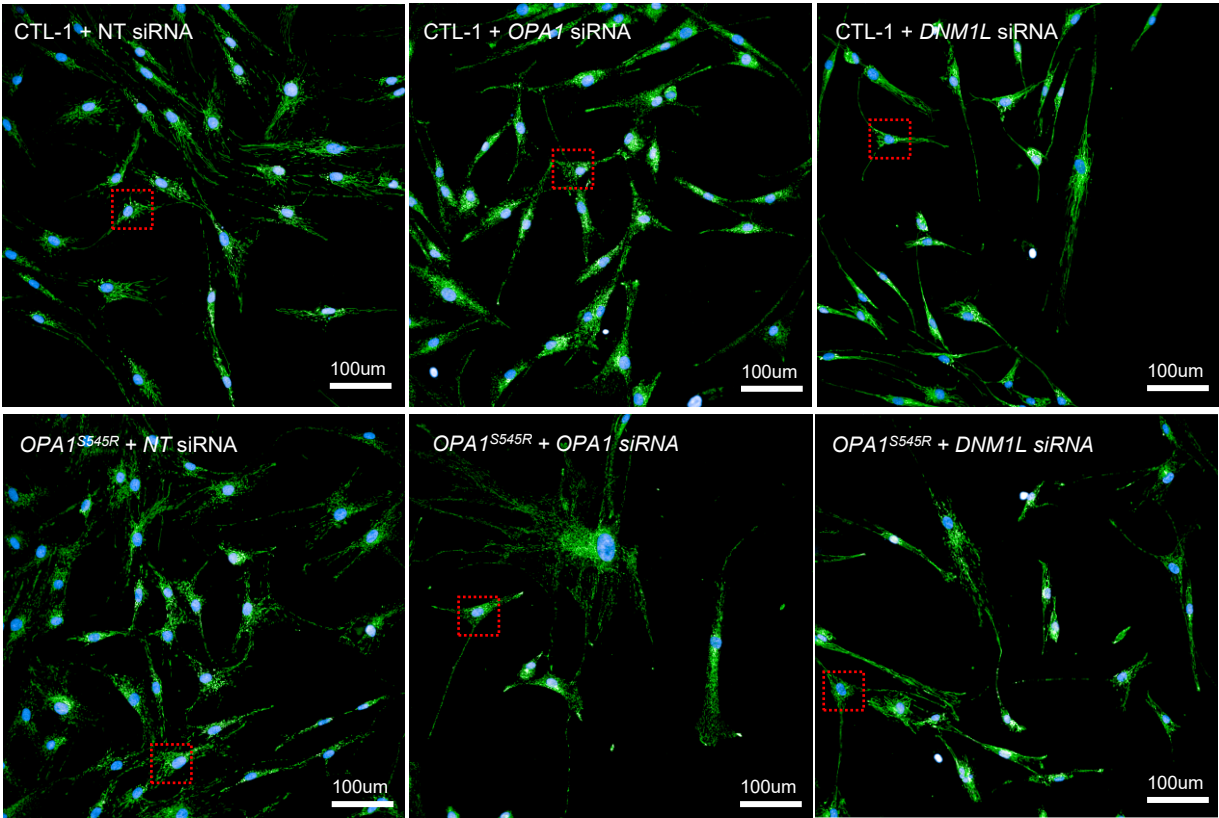

Figure 1F

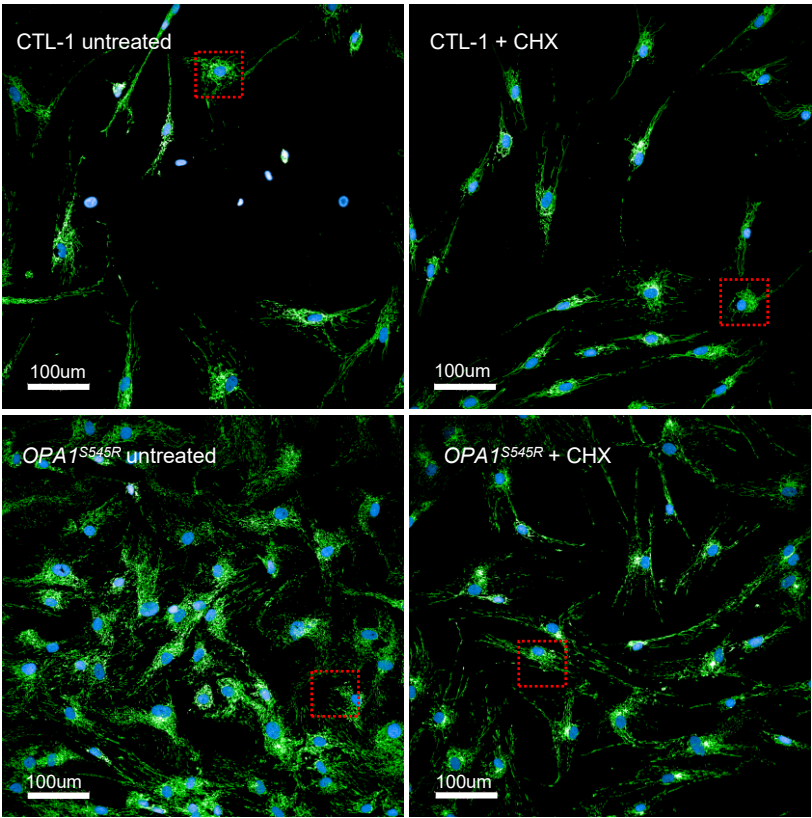

Supplement: Supplementary file 14 — Source Data for Figure 1 [file EMMM-13-e13579-s009.pdf]

Figure 3A

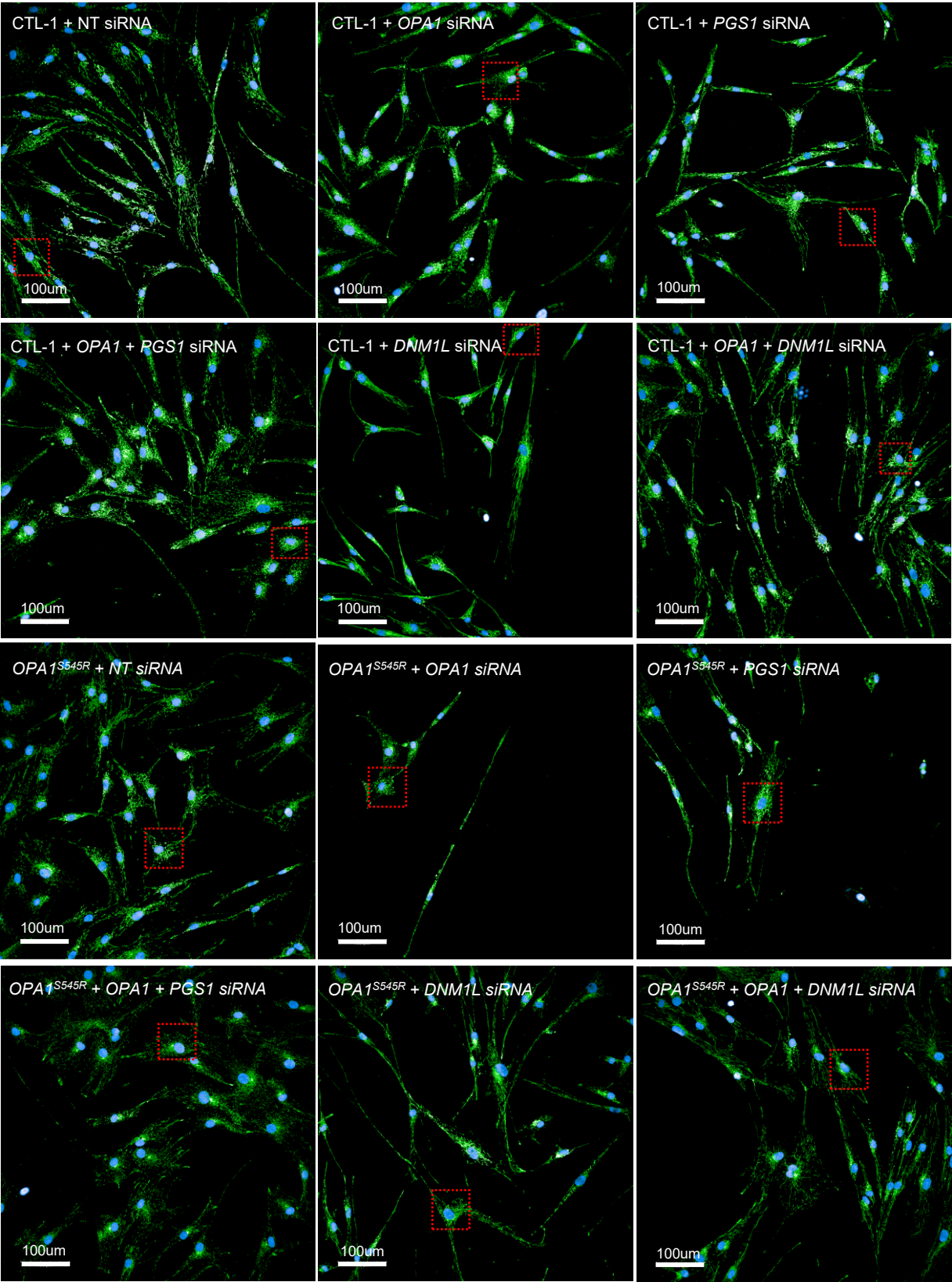

Figure 3C

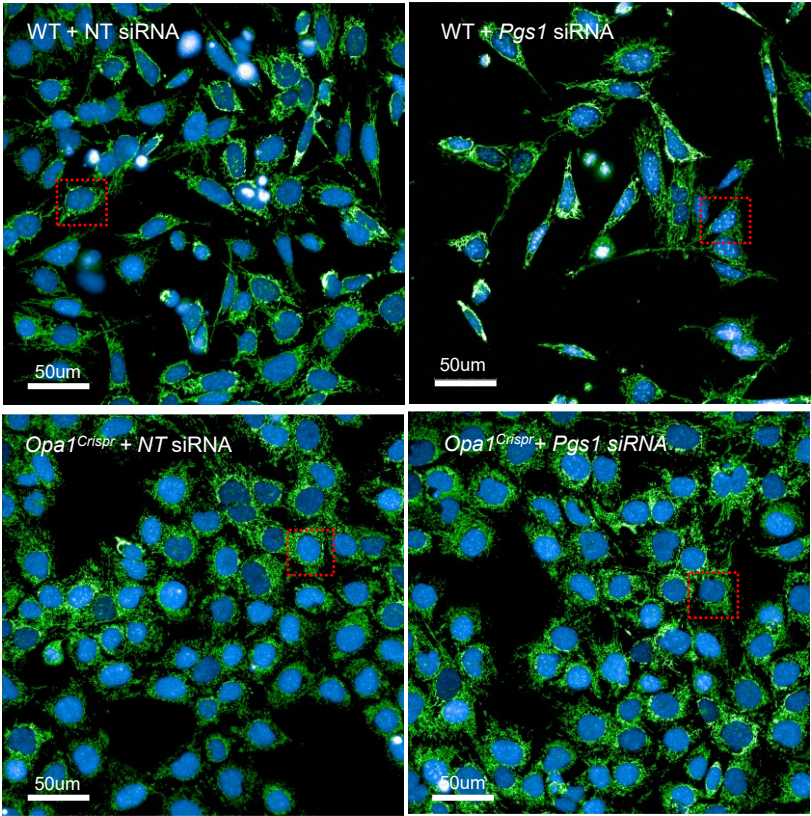

Figure 3E

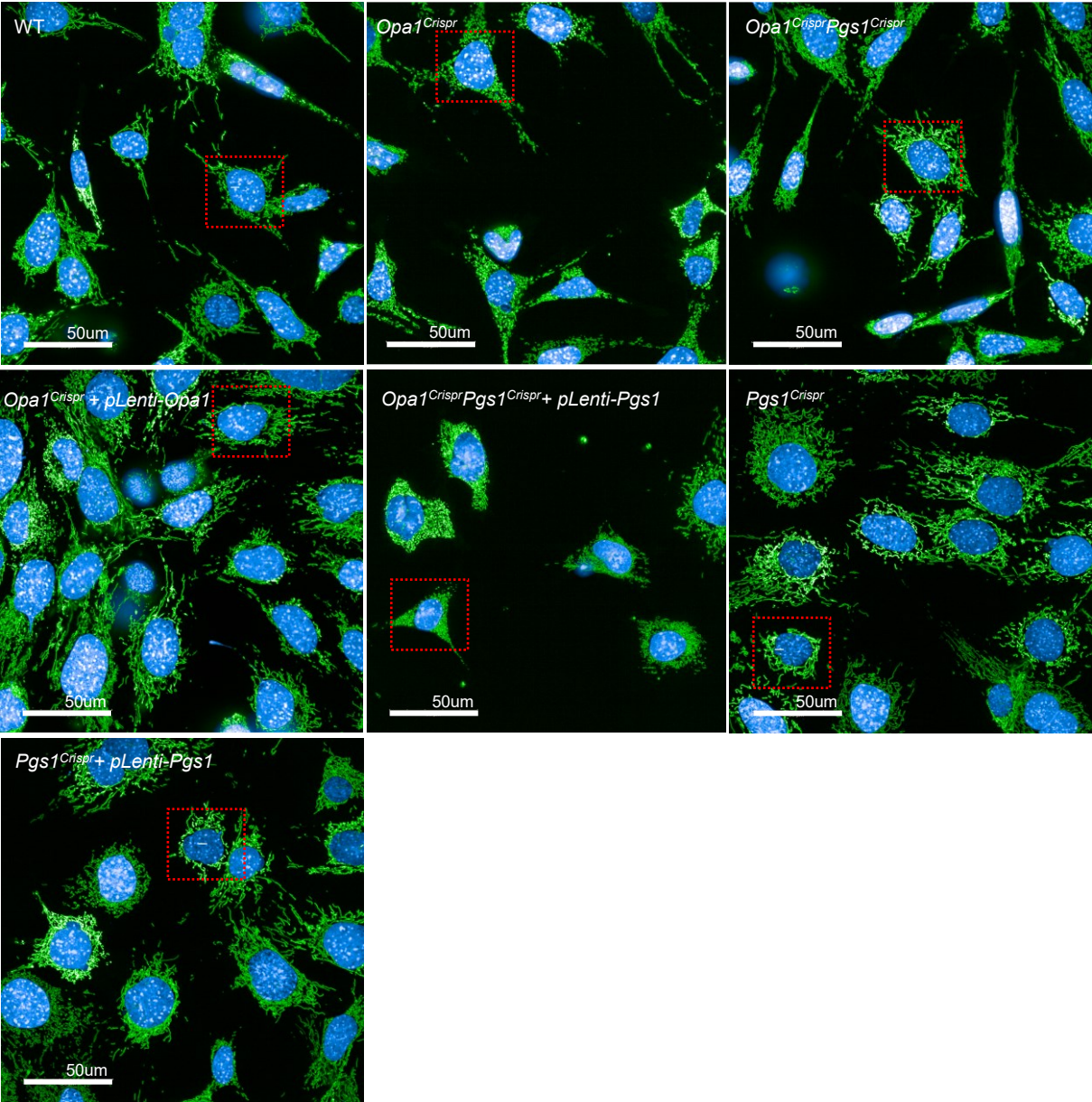

Figure 3G

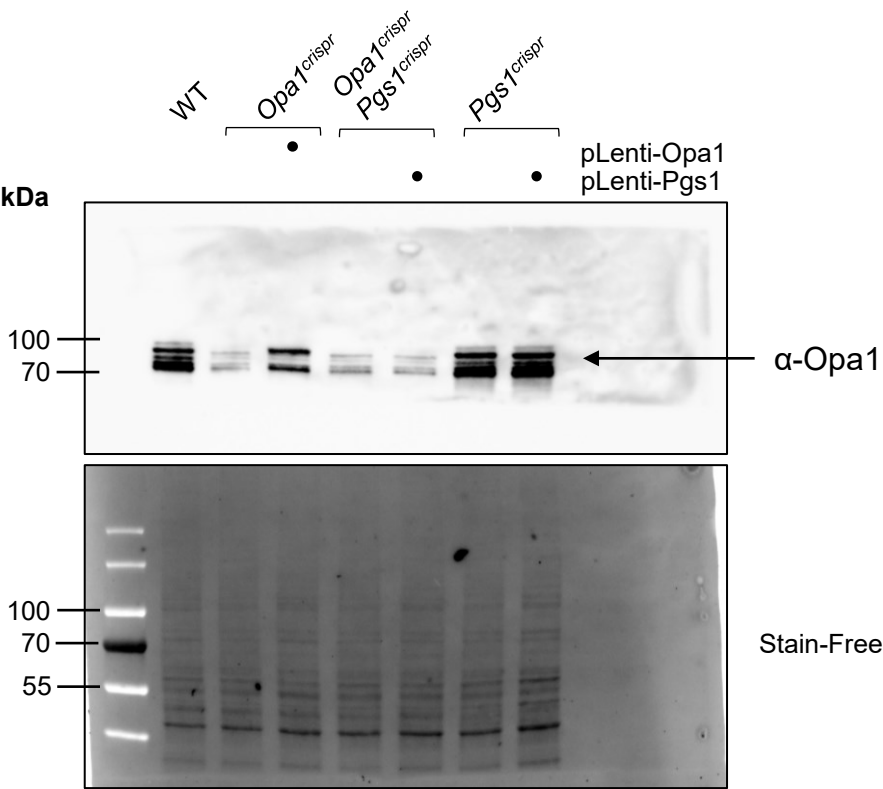

Supplement: Supplementary file 15 — Source Data for Figure 3 [file EMMM-13-e13579-s002.pdf]

Figure 4A

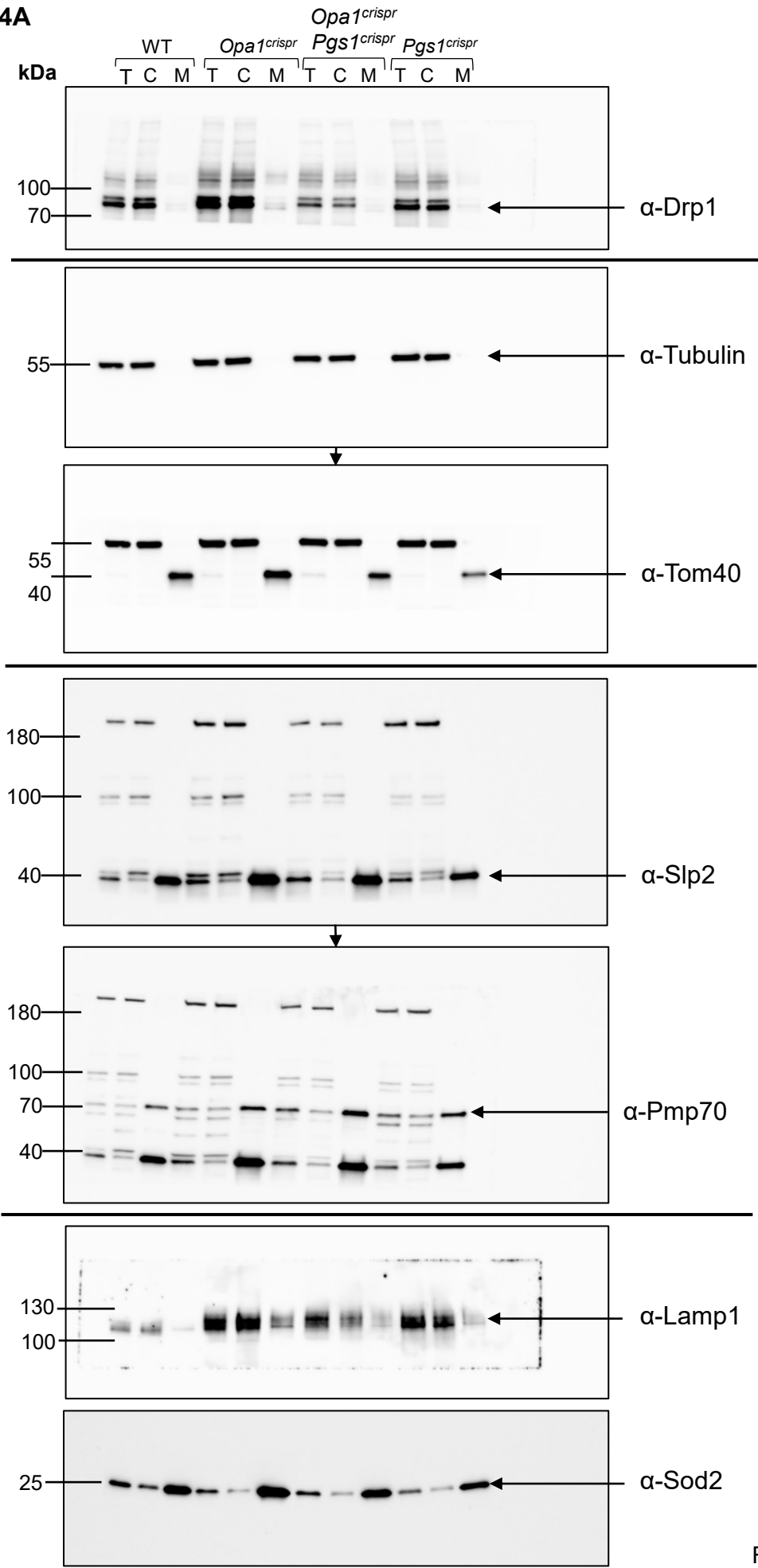

### Figure 4B

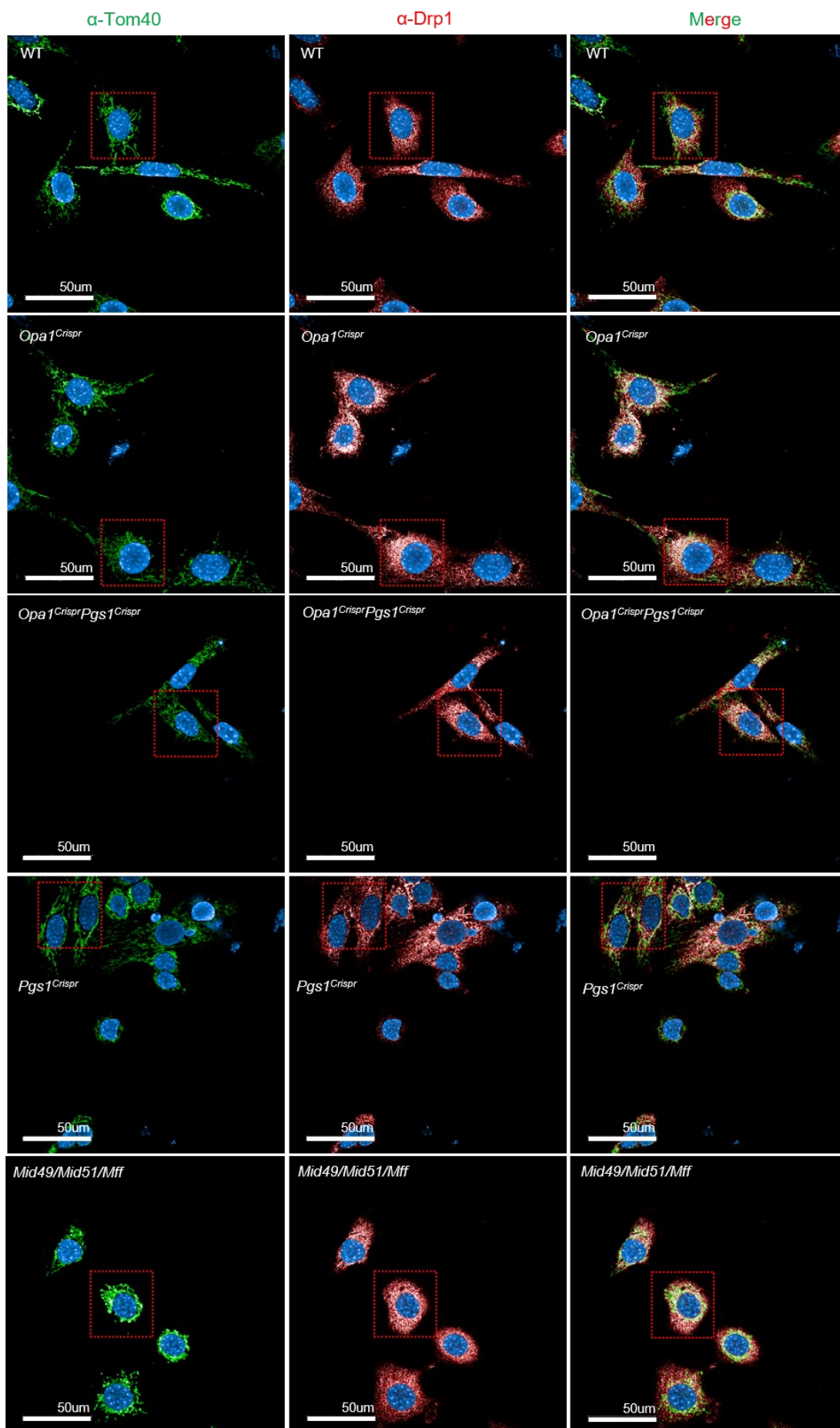



Figure 4C cont.

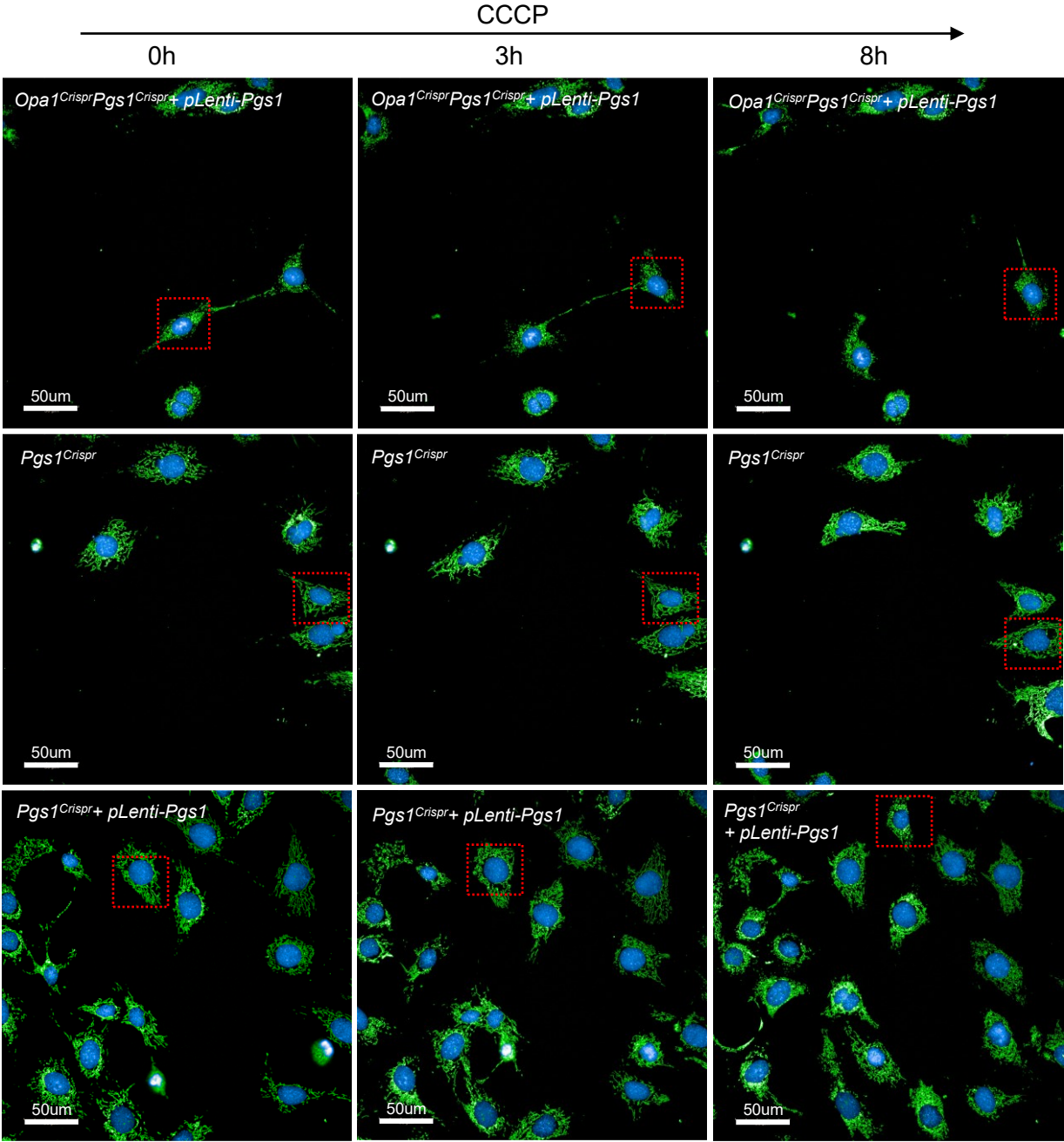

Figure 4F

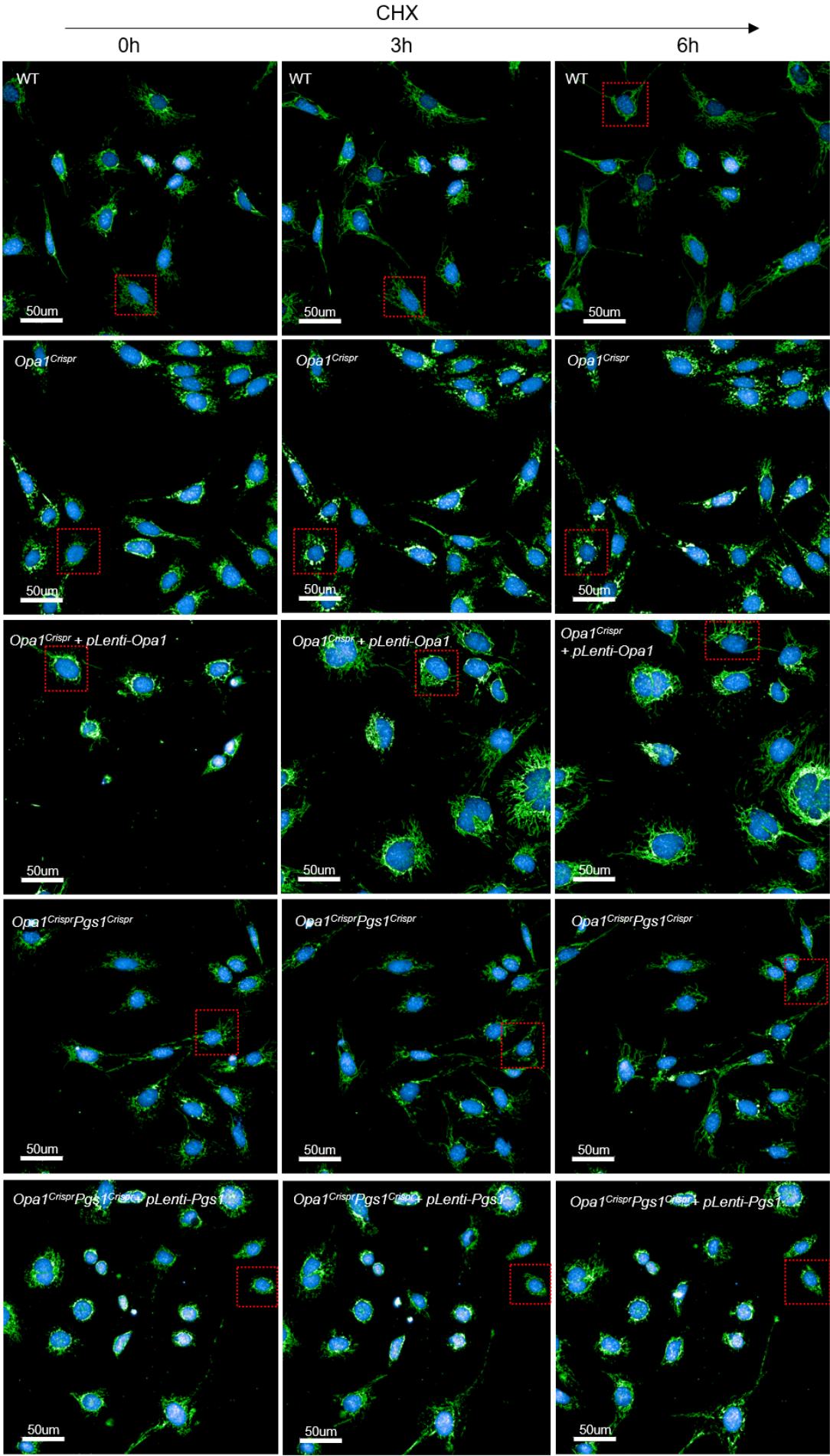

Figure 4\_Source data

Supplement: Supplementary file 16 — Source Data for Figure 4 [file EMMM-13-e13579-s005.pdf]

Figure 5B

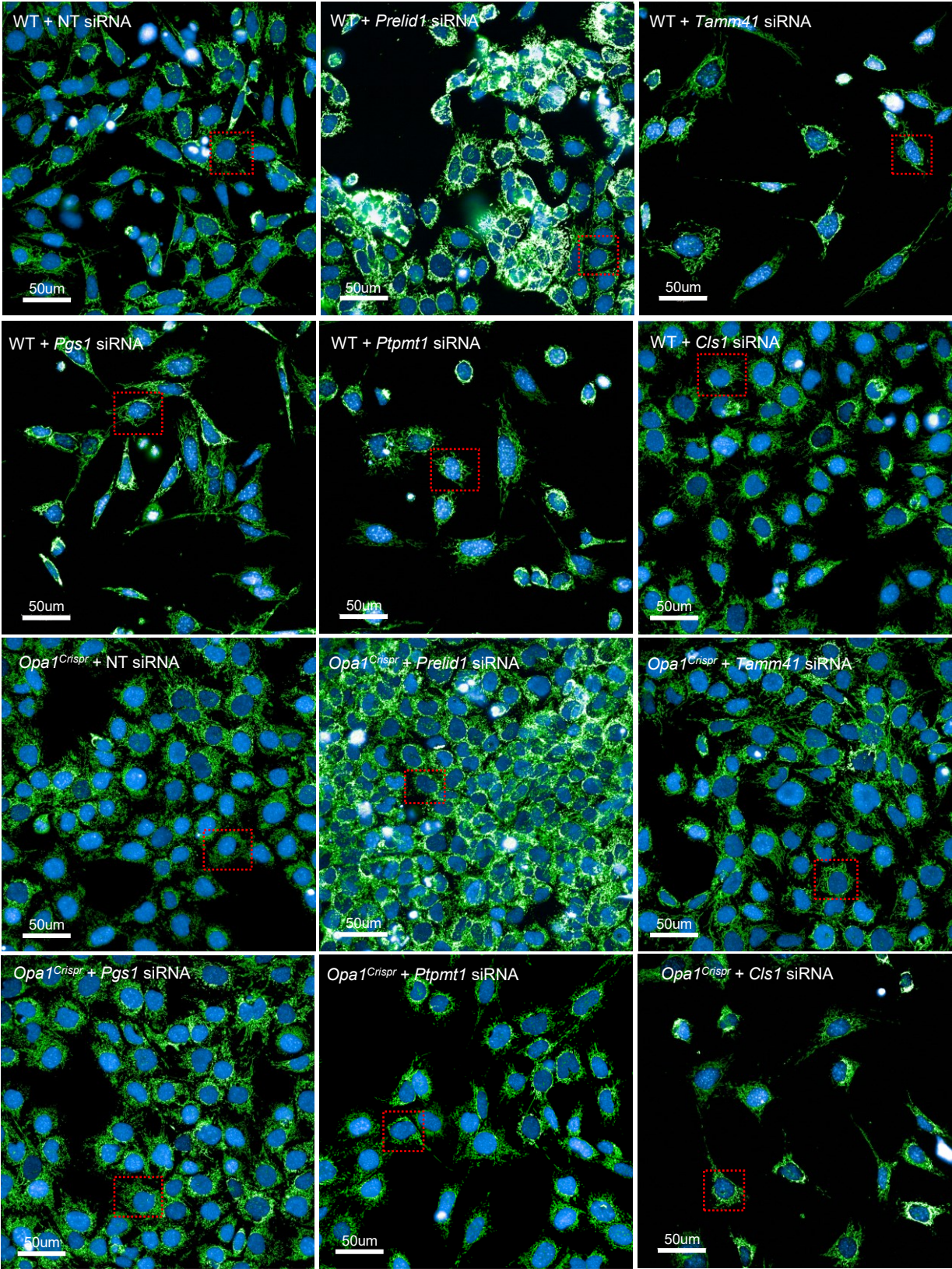

Figure 5G

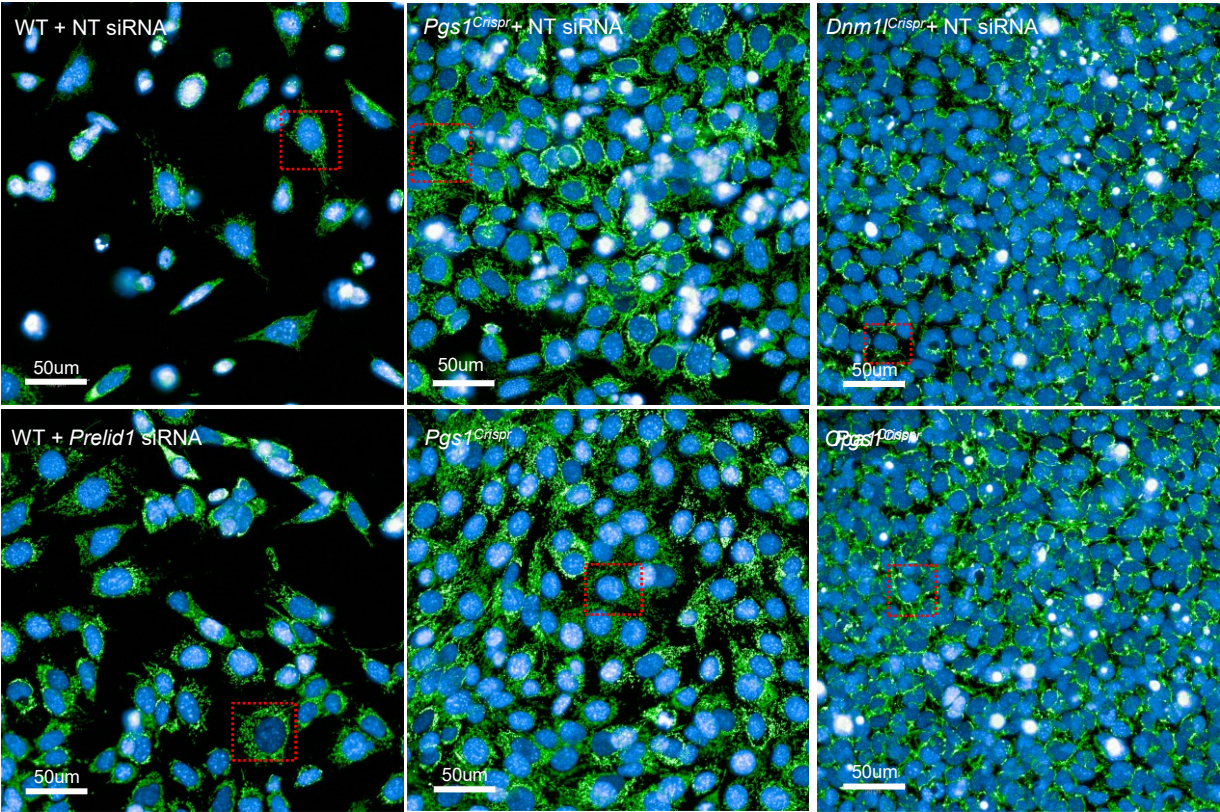

Supplement: Supplementary file 17 — Source Data for Figure 5 [file EMMM-13-e13579-s014.pdf]

Figure 6C

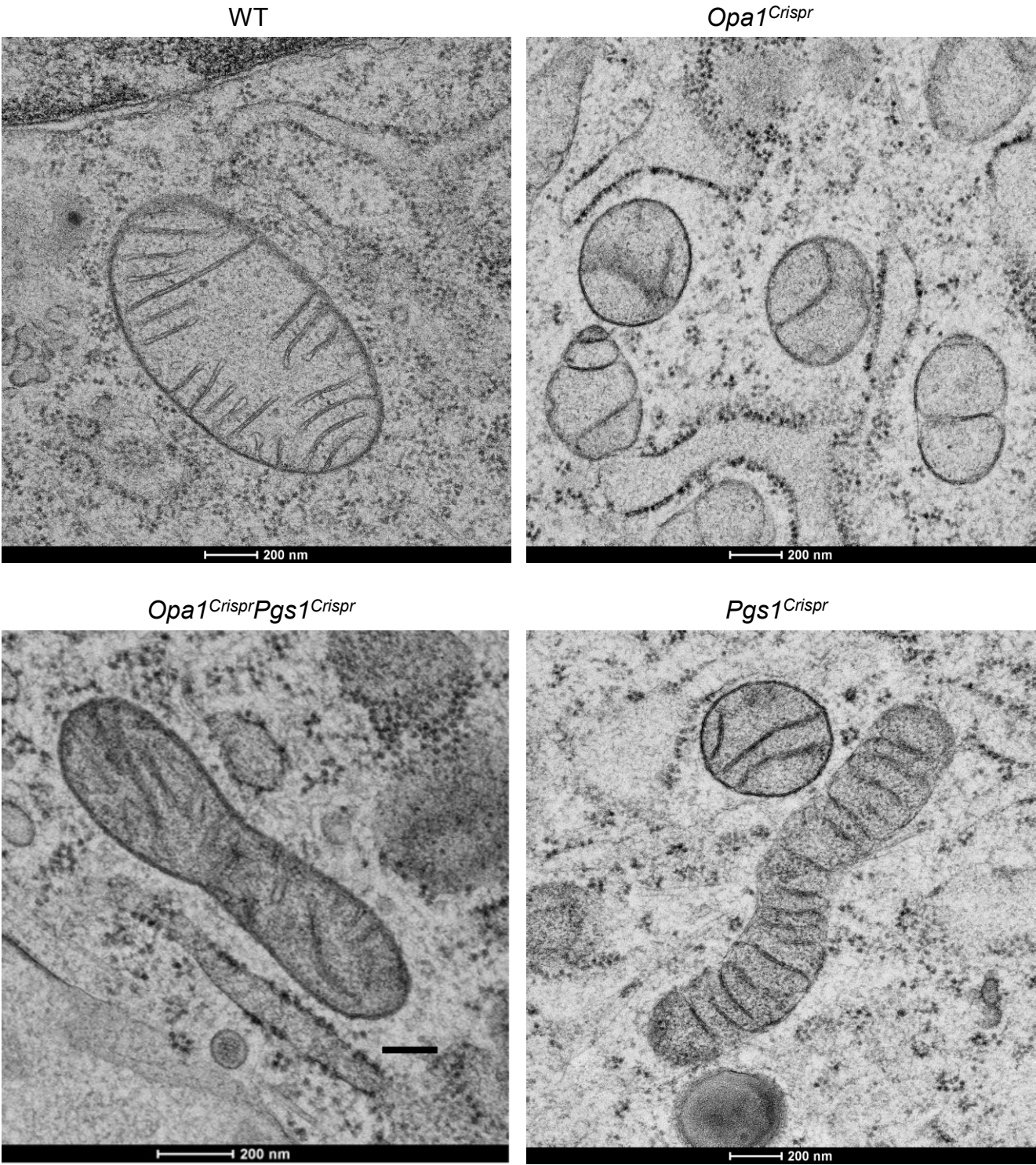

Supplement: Supplementary file 18 — Source Data for Figure 6 [file EMMM-13-e13579-s016.pdf]

Figure 7G

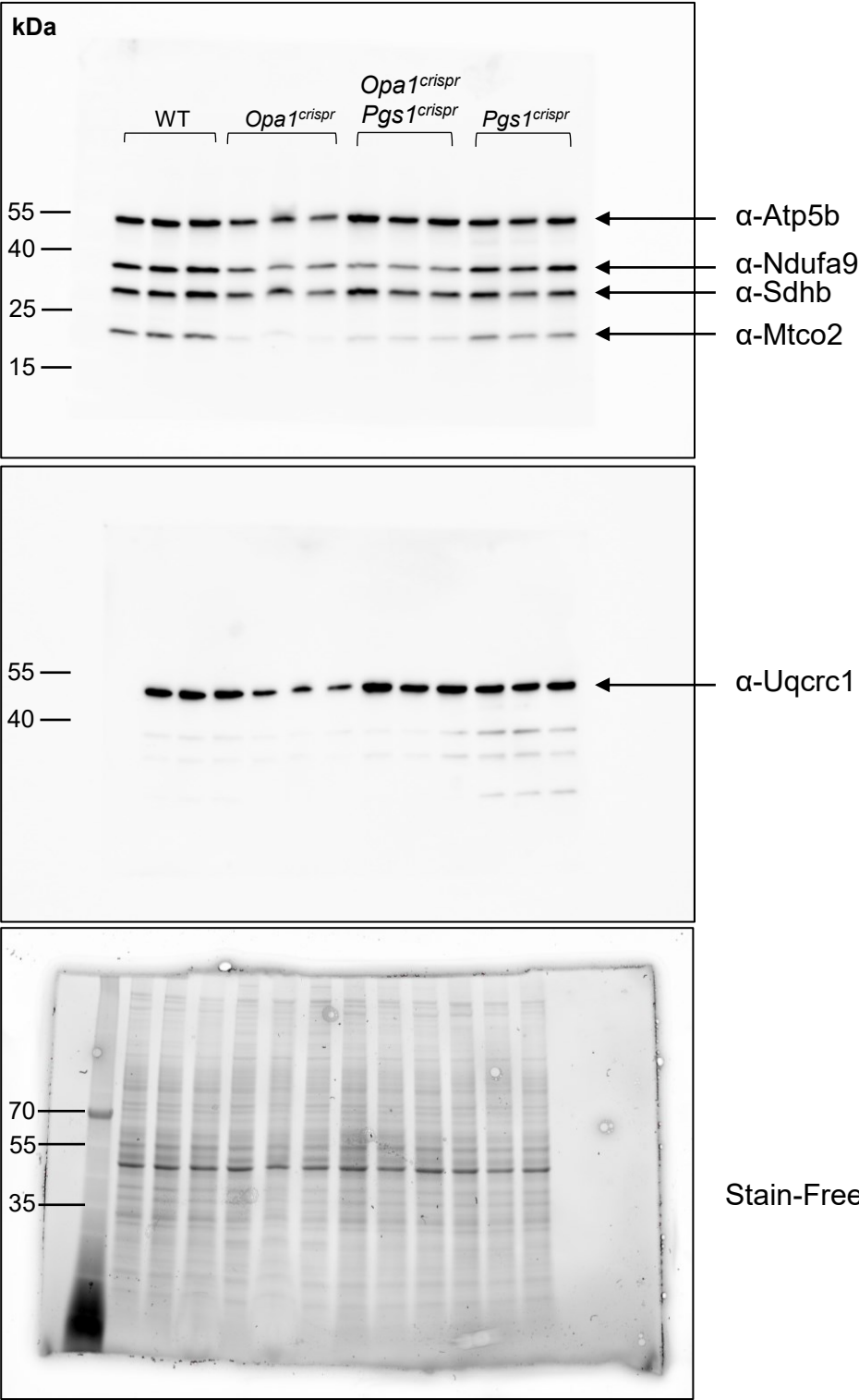

Figure 7G cont.

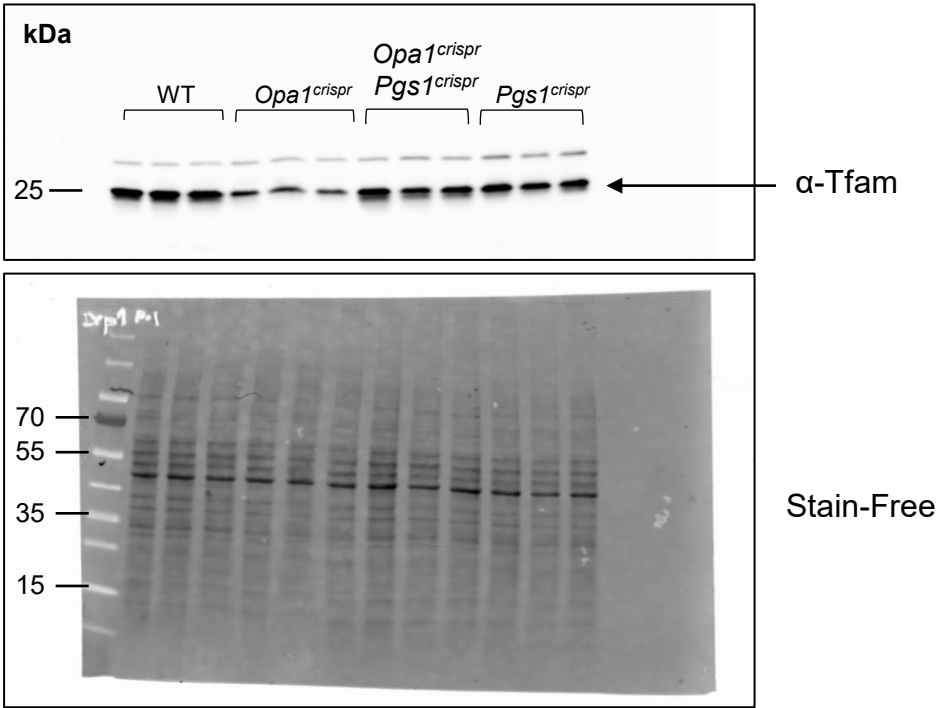

Supplement: Supplementary file 19 — Source Data for Figure 7 [file EMMM-13-e13579-s012.pdf]
